# Belgian endive-derived biostimulant activity in *Arabidopsis*, lettuce, and sweet pepper at different developmental stages, environmental conditions, and application methods

**DOI:** 10.3389/fpls.2026.1852440

**Published:** 2026-07-01

**Authors:** Halimat Yewande Ogunsanya, Christophe Petit, Kris Audenaert, Noémie De Zutter, Danny Geelen

**Affiliations:** 1 Department of Plants and Crops, HortiCell group, Faculty of Bioscience Engineering, Ghent University, Ghent, Belgium; 2 Department of Plants and Crops, Laboratory of Applied Mycology and Phenomics, Faculty of Bioscience Engineering, Ghent University, Ghent, Belgium

**Keywords:** abiotic stress, Belgian endive extract, biostimulant, crop yield, photosynthesis efficiency, plant growth promotion

## Abstract

Belgian endive-derived biostimulant (BEE) has been previously shown to enhance root and shoot growth of *Arabidopsis thaliana* and *Plectranthus esculentus* in *in vitro* culturing conditions. In this study, we evaluated the effect of BEE on *A. thaliana* subjected to abiotic stresses and assessed the translatability of its bioactivity to lettuce (*Lactuca sativa*) and sweet pepper (*Capsicum annuum*) grown in substrate and soil. A first set of experiments tested the impact of BEE on protection during and on recovery after osmotic or salt (NaCl) stress. BEE treatment had little to no restorative effect when plants were exposed to osmotic stress. In contrast, BEE strongly promoted shoot development and leaf health under both standard and NaCl stress conditions. Under mild NaCl stress, BEE enhanced photosynthetic efficiency and chlorophyll content in *Arabidopsis*, whereas it did not significantly alleviate osmotic stress induced by sorbitol. To evaluate its effect under *ex vitro* conditions, BEE was applied via root drenching to substrate-grown *A. thaliana*, lettuce, and sweet pepper. BEE improved leaf greenness and photosynthesis, enhancing *Arabidopsis* rosette development, but did not increase lettuce head weight. In sweet pepper, BEE increased fruit yield and promoted fruit maturation. Under drought stress conditions, however, BEE application did not improve sweet pepper yield. These results indicate that *in vitro* BEE growth promotion is translatable to ex vitro cultivation system and its effect under stress conditions vary depending on the kind of stress. Additionally, BEE is consistent in improving photosynthesis parameters,fruit maturation and ripening.

## 1 Introduction

The increasing global population, projected to reach approximately 9.7 billion by 2050, is placing pressure on agricultural systems to meet the growing demand for food. This pressure necessitates innovative and sustainable approaches to enhance agricultural productivity while minimizing environmental impact. One promising strategy to achieve this goal is the discovery, development, and application of plant biostimulants.

The discovery of biostimulant activity through the screening of (organic) materials often involves treatment of *in vitro* cultured *Arabidopsis* seedlings followed by validation bioassays (Li et al., 2026). Such studies, however, do not provide insight into putative biostimulant activity in horticultural plants, nor whether such activity is preserved under field or substrate cultivation conditions. To evaluate the potential of a biostimulant, studies using economically important species (e.g., sweet pepper) cultivated under commercial growing conditions are necessary (Calvo et al., 2014). Additionally, the effectiveness of biostimulants is often assessed under different environmental stress conditions, including drought stress, salinity stress, and temperature variations, to determine their adaptability and mode of action in real-world farming systems. Such trials provide valuable insights into dosage optimization, application timing, and potential interactions with conventional agricultural practices, ensuring that a biostimulant is both effective and practical for large-scale implementation.

Salt and drought stress are increasingly important challenges in sweet pepper cultivation under greenhouse conditions, where optimal control of environmental factors is essential for high productivity and fruit quality. Despite the controlled environment, water-use efficiency remains a concern, especially as greenhouse agriculture expands in water-limited regions. Drought stress in greenhouses can impair stomatal function, reduce photosynthetic efficiency, and limit biomass accumulation in sweet pepper plants (Dorji et al., 2005). Similarly, salt accumulation in recirculating hydroponic or soilless systems, common in greenhouses, can lead to ionic imbalance, osmotic stress, and nutrient antagonism, ultimately affecting fruit development and yield (Savvas and Gruda, 2018). Plant extract-based biostimulants have been reported to be highly effective in promoting growth in field experiments (Li et al., 2022b). The impact of biostimulant application is often most pronounced under stress conditions (Calvo et al., 2014; du Jardin, 2015; Colla and Rouphael, 2020).

Biostimulants may enhance vegetative and/or reproductive stages (Parađiković et al., 2019). While many commercially available biostimulants exhibit activity across multiple growth stages, identifying the specific developmental stage targeted by a biostimulant can enhance its efficiency. This is because biostimulant responses are influenced by application timing, growth stage, application method, crop species, and environmental conditions (Sible et al., 2021; Andreotti et al., 2022). For instance, biostimulants that specifically promote vegetative growth can be employed during early crop establishment to ensure strong root and shoot development, as demonstrated in studies reporting improved root and shoot growth, seedling performance, leaf area, and biomass following biostimulant application (Ogunsanya et al., 2022; Atero-Calvo et al., 2025). Biostimulants that enhance reproductive growth can be applied during flowering and fruiting stages to improve yield and quality, as reported in a sweet pepper study in which application during pre- and post-transplanting stages improved yield, fruit number, and fruit quality (Zamljen et al., 2024). Likewise, biostimulants that enhance vegetative growth may be particularly beneficial for leafy vegetables such as lettuce, where the crop is grown mainly for leaf biomass and quality, whereas those that promote reproductive development may be more suitable for fruit-bearing crops such as tomato and sweet pepper (Zamljen et al., 2024; Turan et al., 2026). This stage-specific application of biostimulants can lead to more efficient agricultural practices by maximizing crop performance and yield.

Previously, we reported the *in vitro* root- and shoot-growth-stimulating activity of Belgian endive extract (BEE), derived from forced roots, a waste product of Belgian endive (*Cichorium intybus* var. *foliosum*) production (Ogunsanya et al., 2022). In the present follow-up study, we analyzed the impact of BEE across different environmental conditions using the model plant *A. thaliana* and the agriculturally relevant crops lettuce and sweet pepper. These experiments included *in vitro* assays assessing the physiological effects, in addition to indoor and greenhouse experiments. The efficacy of BEE was evaluated under both standard growth conditions and abiotic stress conditions, including salinity stress and drought stress, both of which represent critical challenges to global agricultural productivity. Additionally, while a previous study highlighted the positive effect of BEE on vegetative growth *in vitro* (Ogunsanya et al., 2022), here we explore the impact of BEE on both vegetative and reproductive growth under *ex vitro* conditions. Our findings reveal a highly complex mode of action of BEE on both plant growth and development.

## 2 Materials and methods

### 2.1 Extract preparation

BEE was extracted as previously described (Ogunsanya et al., 2022). Briefly, Belgian endive forced roots were dried, ground, and mixed with water. The resulting mixture was homogenized using an industrial “Stephan apparatus”, which is typically used in food processing for high-speed mixing and heating, at the Food Pilot department of ILVO (Instituut voor Landbouw-, Visserij- en Voedingsonderzoek, Ghent, Belgium). The mixture was homogenized at 80 °C under 100 mbar pressure for 2 h, resulting in a sludge. The sludge was cooled and transferred to 1000 µm and 100 µm vibrating sieves stacked together. The extract obtained after passage through the 100 µm sieve constituted water extract referred to as BEE. The concentrations were extrapolated from the liquid extract. Specifically, 1 mL of BEE was dried in an oven at 80–90 °C until a constant weight was achieved. The dry weight in grams corresponds to 1 mL of BEE, and the desired concentration in grams was calculated to mL volume. For *ex vitro* crop experiments, BEE was prepared by autoclaving at 115 °C and allowed to cool before application to the plants. BEE batch 2020 was used for the *Arabidopsis* osmotic stress experiment, and BEE batch 2022 was used for all other experiments.

### 2.2 *Arabidopsis* experiments

#### 2.2.1 Seed sterilization, media composition, and growth conditions

*A. thaliana* Col-0 wildtype seeds were vapor-phase sterilized and sown on a 12 x 12 cm^2^ plate containing Murashige and Skoog (MS) medium. The MS medium consisted of 1.5 g/L MS basal salt, 0.5 g/L MES buffer (2-(N-morpholino) ethanesulfonic acid), 5 g/L sucrose, and 8 g/L plant agar, adjusted to pH 5.7 with 1 M KOH. Seeds were subjected to stratification in the dark at 5 °C for four days to ensure synchronized germination, followed by 8 h of light exposure in a growth room (100 µmol/m^2^/s intensity, 16 h light/8 h dark photoperiod at 21 °C) and subsequently kept in the dark for 3 days to induce etiolation (Trinh et al., 2018). Seeds transferred to growing substrates did not undergo etiolation and were kept under light conditions for 5 days.

#### 2.2.2 *In vitro* sorbitol osmotic stress assay

After three days of etiolation, *Arabidopsis* seedlings were transferred to treatment plates. The treatment plates consisted of either MS medium (no sorbitol), MS supplemented with 100 mM sorbitol, or MS supplemented with 150 mM sorbitol. Each stress condition was combined with either 0.36 g/L BEE, 0.71 g/L BEE, or no BEE (water control). Ten days after transfer, seedlings were imaged for phenotypic analysis. Primary root length and leaf area were measured using Fiji (ImageJ), while lateral root numbers were counted under a binocular microscope (Olympus SZX9). For the rescue test, seedlings grown on MS plates supplemented with 150 mM sorbitol were transferred to fresh 150 mM sorbitol plates and grown for an additional 7 days under light conditions. Subsequently, they were transferred to rescue MS plates containing either 0.36 g/L BEE, 0.71 g/L BEE, or no BEE. The seedlings were left in the rescue plates for 3 days and subsequently analyzed for their phenotypes.

#### 2.2.3 *In vitro* salinity stress assay

The true leaf development assay was adapted from Li et al. (2022a) to evaluate the effects of BEE on early vegetative growth under salt stress. *A. thaliana* seeds were pre-selected using a binocular microscope (Olympus) to ensure viability and reduce variability. Sterilized seeds were then sown on MS medium supplemented with either 0 mM or 100 mM NaCl, with or without 0.1 g/L of BEE. The 100 mM NaCl concentration was used to induce mild salinity stress. Following sowing, seeds were stratified in darkness at 5 °C for four days, after which they were incubated horizontally in a growth room (100 µmol/m^2^/s light intensity, 16 h light/8 h dark photoperiod at 21 °C) for 10 days. Each treatment consisted of three replicate plates containing 50 seeds per plate to further minimize seed-to-seed variability. Germination was assessed by calculating the proportion of seeds exhibiting visible radicle emergence following testa rupture relative to the total number of sown seeds. Development of the first true leaves was monitored through imaging seedling six days after sowing (DAS) using a binocular microscope (Olympus SZX9) equipped with a 10x eyepiece (WHS10X-H/22) and a 1x objective lens (DFPLAPO1x-2). The microscope was operated at a zoom setting of 2.0x, corresponding to a total magnification of 20X. The percentage of true leaf emergence was calculated as the number of germinated seeds that developed true leaves divided by the total number of germinated seeds. Ten days after sowing, plant images were captured with an RGB Nikon camera, and leaf area was quantified using ImageJ. To complement morphological observations, plant health and development were evaluated using a multispectral imaging platform (Phenovation, Wageningen, The Netherlands). The overall plant health status was evaluated using chlorophyll fluorescence (Fv/Fm), a proxy for the efficiency of photosynthesis (Baker, 2008). Chromophore-based proxies were included to evaluate the chlorophyll and anthocyanin content of the *Arabidopsis* plants. Specifically, the chlorophyll index (ChlIdx) (Gitelson et al., 2003)) and the modified anthocyanin reflectance index (mARI) (Gitelson et al., 2009)) were calculated. Detailed descriptions of these spectral parameters are provided in **Supplementary Table 1** of the **Supplementary Materials**.

#### 2.2.4 *Arabidopsis* growth on substrate, treatments, and measurements

*A. thaliana* Col-0 wild-type seeds were germinated as described in section 2.2.1. Seedlings were then transferred to Jiffy peat pods (Jiffy Products International AS, Norway) and covered with plastic film for 3 days. The seedlings were treated and left to grow in a growth room under a 16 h light/8 h dark photoperiod (light density 70 µm m^-2^ s^-1^, 21 °C). Two forms of BEE treatment were employed: heat-treated (autoclaved; Auto_BEE) and non-autoclaved (NA_BEE). The treatments were applied using two methods: root drenching and shoot spraying (**Table 1**). Each treatment contained 18 plants. Non-autoclaved or autoclaved BEE was applied at concentrations of 0.18 g/L or 0.36 g/L, with water serving as the control. Treatments were applied 5 days after transfer to the Jiffy pods. The root treatment was performed by drenching the trays containing the plants with the respective treatment solutions (approximately 1–2 L). The shoot treatment was performed by spraying each plant with approximately 3 mL of the respective treatment 5 days after transfer to the Jiffy pods (i.e., 14 DAS). The plants were subsequently watered two to three times per week with fertilizer (Wuxal 8-8-6). The leaf area was used as a vegetative parameter. At 23 DAS, the leaf area phenotype of each plant was evaluated. A Nikon RGB camera was used to take pictures of the trays containing the plants for leaf area scoring. The scoring of the leaf area was quantified using Fiji.

**Table 1:** Overview of the conditions used for BEE application in *Arabidopsis*, lettuce, and sweet pepper experiments.

No	Plant species	Stress	*In vitro* / *Ex vitro*	BEE application method	BEE concentration	BEE Autoclaved or non-autoclaved	Growing substrate	Developmental stage analyzed
1	*A. thaliana*	Osmotic	*In vitro*	*In vitro* plate	0.36 g/L & 0.71 g/L	Autoclaved	MS media	Vegetative
2	*A. thaliana*	Salinity	*In vitro*	*In vitro* plate	0.1 g/L	Autoclaved	MS media	Vegetative
3	*A. thaliana*	None	*Ex vitro* (Growth room)	Root applied & shoot applied	0.18 g/L & 0.36 g/L	Autoclaved and non-autoclaved	Peat	Vegetative
4	*L. sativa*	None	*Ex vitro* (Growth room)	Root applied	0.18 g/L & 0.36 g/L	Autoclaved	Peat	Vegetative
5.1	*C. annuum*	None	*Ex vitro* (Greenhouse)	Root applied	2 g/L	Autoclaved	Potting soil	Vegetative and reproductive
5.2	*C. annuum*	Drought	*Ex vitro* (Greenhouse)	Root applied	2 g/L	Autoclaved	Potting soil	Vegetative and reproductive
6	*C. annuum*	None	*Ex vitro* (Growth room)	Root applied	2 g/L	Autoclaved	Rockwool slab	Vegetative and reproductive
7.1	*C. annuum*	None	*Ex vitro* (Greenhouse)	Root applied	2 g/L	Autoclaved	Potting soil	Vegetative and reproductive
7.2	*C. annuum*	Drought	*Ex vitro* (Greenhouse)	Root applied	2 g/L	Autoclaved	Potting soil	Vegetative and reproductive

### 2.3 Lettuce growth, treatments, and measurements

Lettuce (*Lactuca sativa* var. Expertise, Rijk Zwaan) seeds were germinated directly on pre-wetted Jiffy peat pods and kept in a growth chamber under controlled conditions: 18 °C, 60–70% relative humidity, and 200–220 μmol/m^-2^/s light intensity with a 16 h photoperiod. Uniformly grown seedlings were selected and transferred to new trays at 10 DAS (**Supplementary Figure 1**). Six seedlings were used per tray per treatment, with three technical replicates. The lettuce seedlings were treated with 0.18 g/L or 0.36 g/L BEE, or left untreated as controls (**Table 1**). Plants were evaluated at 41 DAS. Fresh shoot weight was quantified, after which samples were dried in an oven at 70 °C for 24–48 h for dry weight quantification. Polypen RP 410 UVIS (PSI, Photon Systems Instruments, Czech Republic) was used to measure the leaf indices. Leaf reflectance (R) was calculated as I/I_0_, where I represents the sample reflectance signal and I_0_ represents the reflectance from a Spectralon calibration standard (Van de Velde et al., 2023). The Normalized Difference Vegetation Index (NDVI), Gitelson and Merzlyak Index 1 (GM1), Carotenoid Reflectance Index 1 (CRI1), Anthocyanin Reflectance Index 1 (ARI1), Greenness Index (G), and Carter Index 1 (Ctr1) were recorded. Detailed descriptions of these indices are summarized in **Supplementary Table 1**.

### 2.4 Sweet pepper growth, treatments, and measurements

The sweet pepper cultivar *Capsicum annuum* ‘MADURO F1’ (F1 hybrid) seeds were sourced from Enza Zaden (Enkhuizen, The Netherlands). Sweet pepper seeds were immersed in water at 40 °C to break dormancy, after which they were germinated on wet Whatman paper in a Petri dish. The Petri dish containing the seeds was wrapped in aluminum foil and placed in a growth chamber at 25 °C in the dark for 3 days. Germinated seedlings were transferred to Jiffy peat pods (Jiffy Products International AS, Norway) and incubated in a growth room under 100–110 µmol/m^2^/s light intensity and a 16 h light/8 h dark photoperiod at 25 °C for 25 days. Seedlings were subsequently transplanted into pots containing potting soil (Experiments 5 and 7; **Table 1**) or rockwool blocks (Experiment 6; **Table 1**) and transported to a greenhouse (at ILVO) or a growth room (at the Faculty of Bioscience Engineering, Ghent University, Belgium) under controlled temperature (25–28°C), light intensity (220–260 µmol/m^2^/s), and humidity (approximately 60%). The plants were uniformly irrigated once per day and maintained under a 16 h photoperiod. In the greenhouse experiments, the temperature ranged between 24 °C and 28 °C, and a dripping irrigation system was used to irrigate the plants daily. The experiment was conducted with two main treatment groups: the BEE-treated group and the control group. In the BEE group, the sweet pepper plant received BEE treatment as a supplement, while the control group was not supplemented but received water instead. A concentration of 2 g/L of BEE was applied to the plants, with 50 mL administered per plant (**Supplementary Table 2**). The BEE treatment was applied via root application one week before the projected onset of flowering. The control plants were given water without supplements. In drought-stressed plants, automatic irrigation was stopped for 3 days in Experiment 7.2 and for 5 days in Experiment 6.2, until the plants exhibited slight wilting and the soil water content reached ≤ 30%. In Experiment 7.2, BEE was applied to the roots at the end of the drought period, while in Experiment 6.2, BEE was applied 15 days before drought induction (**Supplementary Figure 1**).

Data were collected to evaluate the effects of BEE on key growth parameters and yield components in sweet pepper. These include plant height (cm), chlorophyll content, number of fruits per plant, and fruit yield. The plant height was measured from the base to the top of the plant. The chlorophyll content was measured using a DUALEX meter (ForceA, France) to estimate leaf greenness and photosynthetic potential. The DUALEX measures the light transmitted through the leaf at specific wavelengths to estimate the chlorophyll content as an absolute value in µg/cm^2^. Three young leaves from a branch of each plant were measured, and each leaf was assessed at three different spots. The number of fruits per plant was recorded to evaluate the effect of BEE on fruit production. Total fruit yield per plant was recorded as the fresh weight (kg) of all fruits harvested at the end of the experiment. All data were collected at the end of the experiment: 116 DAS for Experiments 5 and 7, and 97 DAS for Experiment 6 (**Supplementary Figure 1**).

The release of ethylene was measured in sweet pepper plants 12 h, 24 h, and 48 h after treatment with BEE. A control measurement (0 h) was taken before the BEE treatment. One young leaf per plant of sweet pepper was detached and placed in a 7 mL glass vial (Supelco). The glass vial was left open for 15 min to allow the wound-induced ethylene burst associated with leaf detachment to dissipate. The glass vials were then sealed with screw caps fitted with PTFE/silicone septum (Supelco) and incubated for 4 h at 25 °C in the dark to allow ethylene accumulation in the headspace of the vial. After incubation, 2 mL of the headspace was collected using a gastight syringe (Supelco) and analyzed using a gas chromatograph (GC, Trace 1300, Thermo Scientific, Waltham, MA, USA) equipped with a flame ionization detector. Calibration was performed using ethylene standards of 0.4 PPM (parts per million) and 1 PPM. Subsequently, the leaves in the vial were dried in an oven at 80 °C, and their weight was used to calculate the ethylene content.

### 2.5 Statistical analyses

Data manipulation and visualization were performed using Python (version 3.2). The pandas library (McKinney, 2011) was used for data preprocessing, while NumPy (Harris et al., 2020) was used for numerical computations. Data visualization was carried out using Matplotlib (Hunter, 2007) and Seaborn (Waskom, 2021). Data were visualized using boxplots with individual data points overlaid. Boxes represent the interquartile range (IQR) with the median, and whiskers indicate the most extreme values within 1.5 x IQR of the first and third quartiles. For datasets with a low number of observations, bar plots displaying the mean ± standard deviation with individual data points overlaid were used instead. Bar plots were also utilized for all graphs within figures where the use of boxplots was not appropriate. For count data, distribution plots were generated, with percentage distributions indicated directly on the plots. Normality and homoscedasticity were assessed using the Shapiro–Wilk and Levene’s tests, respectively, to check if the data were normally distributed. Data that failed the normality test were subjected to non-parametric testing using the Krustal-Wallis test. Otherwise, parametric analyses were performed using analysis of variance (ANOVA). Statistical analyses, including ANOVA and t-tests, were conducted using SciPy (Virtanen et al., 2020) and/or Pingouin (Vallat, 2018). A value of *p* < 0.05 was considered statistically significant. *Post-hoc* multiple comparisons were performed in SciPy using Sidak’s, Tukey’s, or Dunnett’s multiple comparison tests, as appropriate. All scripts and analyses conducted in Python were executed in a JupyterLab environment to ensure reproducibility.

## 3 Results

### 3.1 Effect of BEE on *in-vitro* grown *Arabidopsis* under abiotic stress

#### 3.1.1 Osmotic stress

**Figure 1:**
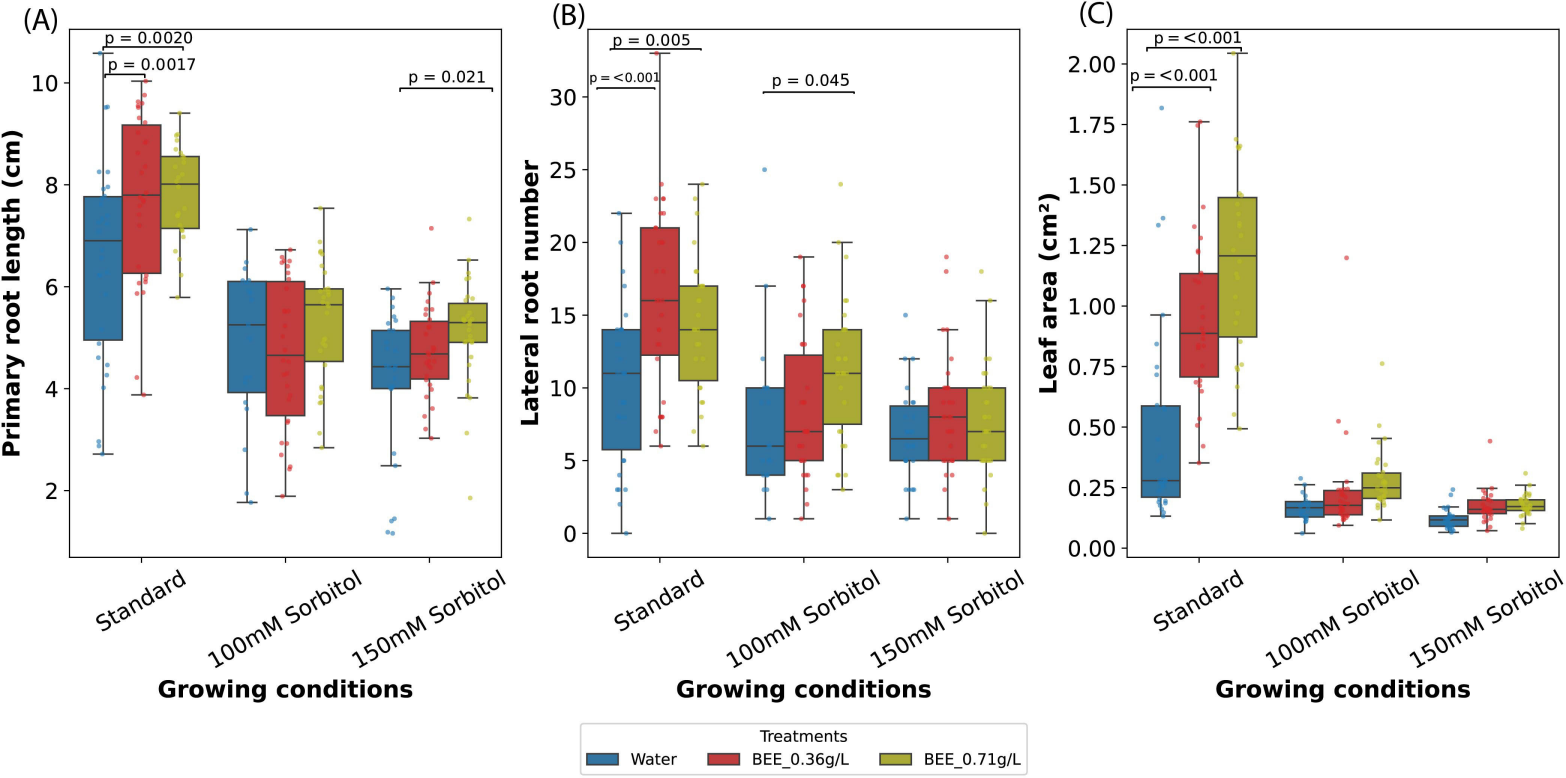
Effect of BEE on root and shoot growth in sorbitol-treated *Arabidopsis* plants. **(A)** primary root length, **(B)** lateral root number, and **(C)** projected leaf area. Data represent the average of three biological and ten technical replicates per bar (30 seedlings in total, 10 per replicate). Significant differences (*p* < 0.05) between the control (Water) and treatments (0.36 g/L or 0.71 g/L of BEE) within each growth condition were determined by two-way ANOVA followed by Sidak’s *post-hoc* test, and are indicated in the figure.

In a first series of experiments, osmotic stress mitigation was assessed by adding BEE at final concentrations of 0.36 g/L and 0.71 g/L to plants grown in the presence of 100 and 150 mM sorbitol. In line with previous observations (Ogunsanya et al., 2022), the primary root length (PRL) significantly increased following BEE treatment in non-stressed plants compared with the control (**Figure 1A**). The application of 150 mM sorbitol inhibited primary root growth, an effect that was partially restored by adding 0.71 g/L BEE (**Figure 1A**; *p* = 0.021). A similar trend was observed in plants treated with 100 mM sorbitol, although the effect was not significant (**Figure 1A**; *p* = 0.249). The lateral root (LR) number was lower in sorbitol-treated plants compared with the control (**Figure 1B**). Application of 0.71 g/L BEE significantly increased lateral root formation under mild osmotic stress (100 mM sorbitol; *p* = 0.045), whereas no significant effect was observed under high osmotic stress (150 mM sorbitol; *p* = 0.072). The application of both 0.36 g/L and 0.71 g/L BEE strongly stimulated leaf growth under standard growing conditions (**Figure 1C**). Leaf area was strongly inhibited by sorbitol treatments and was not significantly restored by BEE supplementation (**Figure 1C**; *p* = 0.245–0.706). These experiments showed that BEE has limited capacity to protect plants against osmotic stress.

In a second set of experiments, the capacity of BEE to promote recovery following osmotic stress was analyzed. *Arabidopsis* plants subjected to 150 mM sorbitol for 7 days were transferred to sorbitol-free medium supplemented with either 0.36 g/L or 0.71 g/L BEE. Rescued plants exhibited significant recovery in primary root length and lateral root number compared with non-rescued plants. Leaf area was likewise greater in rescued plants than in non-rescued controls (**Supplementary Figure 2**). BEE supplementation did not produce significant differences in any of these phenotypes compared with the control medium. Overall, BEE enhanced root elongation, leaf area, and lateral root development under standard conditions, but its effectiveness was reduced under osmotic stress (**Supplementary Figure 3**).

#### 3.1.2 Salinity stress

**Figure 2:**
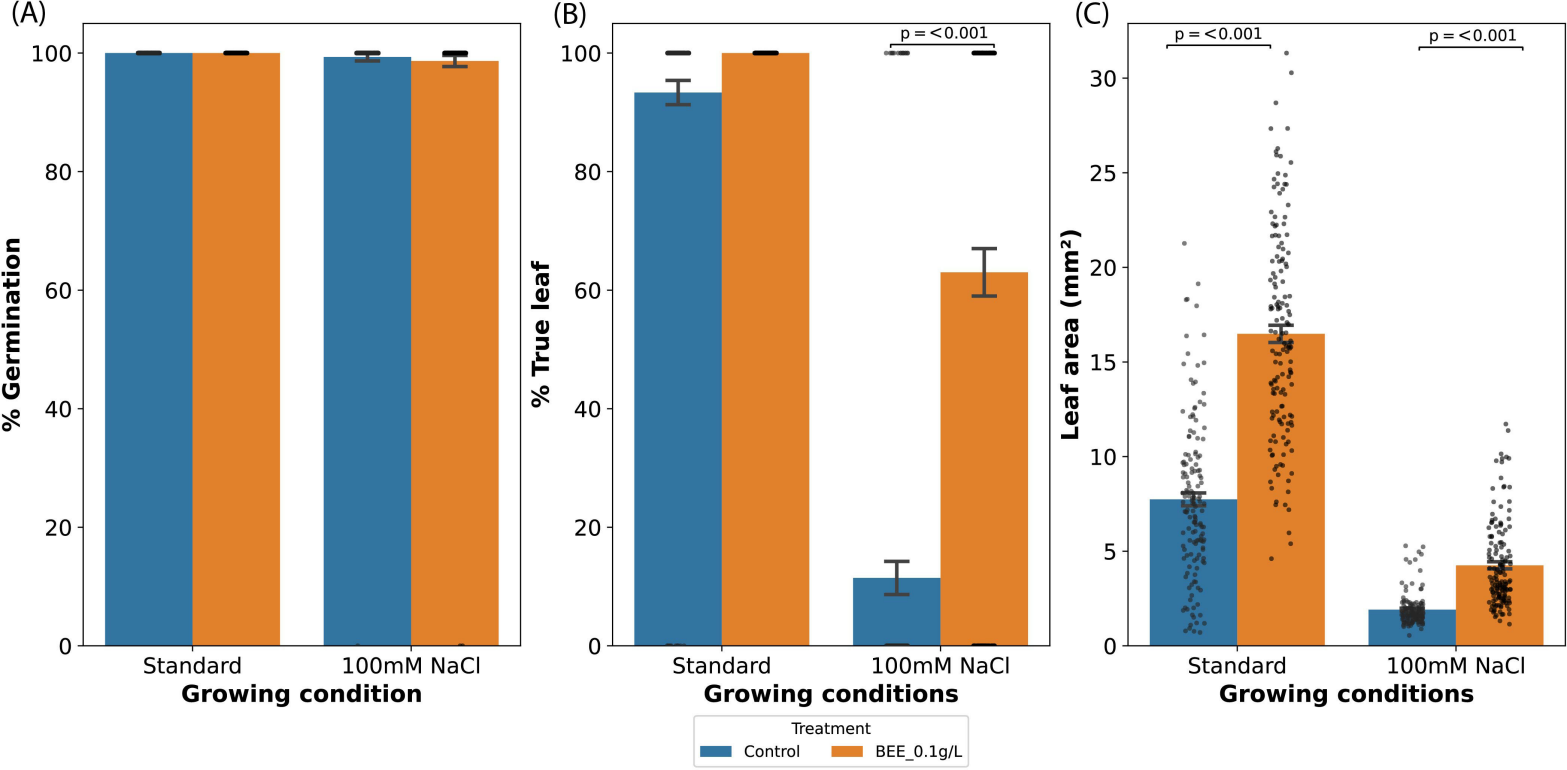
Growth parameters of *Arabidopsis* treated with BEE under standard and 100 mM NaCl growth conditions. Shown are the effects on germination rate **(A)**, true leaf emergence at 6 DAS **(B)**, and leaf area at 10 DAS **(C)**. *p*-values indicate significant differences (p < 0.05, n = 150) between control and BEE treatment in respective groups (growth condition) according to two-way ANOVA followed by Tukey’s HSD test.

To examine salinity stress, *Arabidopsis* was cultured in the presence of 100 mM NaCl. Salinity reduced germination from 100% to 98.67%. Germination was reduced to 98.67% in BEE-treated seeds while it reduced to 99.33% in control seeds (**Figure 2A**). A chi-square test revealed no significant association differences between BEE treatment and germination rate under either growing conditions (χ² = 3.685, df = 3, *p* = 0.298), with germination ranging from 148 to 150 seeds out of 150 seeds across treatments. At 6 DAS, the percentage of true leaves was calculated as the number of emerged first “true” leaves per lot of germinated seeds. Under standard growth conditions, 100% of BEE-treated seedlings developed true leaves compared with 93.33% of seedlings grown in the control medium. The growth stimulation effect was more pronounced under 100 mM NaCl, with 61.33% of BEE-treated seedlings developing true leaves compared with only 11.33% in the control. At 10 DAS, leaf area was measured to evaluate vegetative growth (**Figure 2C**). Under standard growth conditions, BEE-treated plants exhibited a mean leaf area of 16.49 mm^2^, significantly larger than the control (7.74 mm^2^). Similarly, under 100 mM NaCl, leaf area was reduced across all treatments; however, BEE-treated plants showed a significantly larger mean leaf area (4.23 mm^2^) than control plants (1.90 mm^2^). The effect of BEE on leaf area was greater in non-stressed plants, likely reflecting the higher baseline performance of unstressed plants compared to stressed plants. When expressed as the relative change (ratio of +BEE/-BEE), the effect of BEE was highly similar under both conditions (2.23 vs 2.13, respectively; **Supplementary Figure 4**), indicating a consistent proportional effect on leaf area irrespective of stress status. In contrast, the +BEE/-BEE ratio for the emergence of the first true leaf was highly elevated under salt stress (5.50; **Supplementary Figure 4**) but absent under standard conditions (1.07). Together, these results demonstrate that BEE treatment enhances *Arabidopsis* seedling growth under both standard and mild NaCl salt stress conditions.

**Figure 3:**
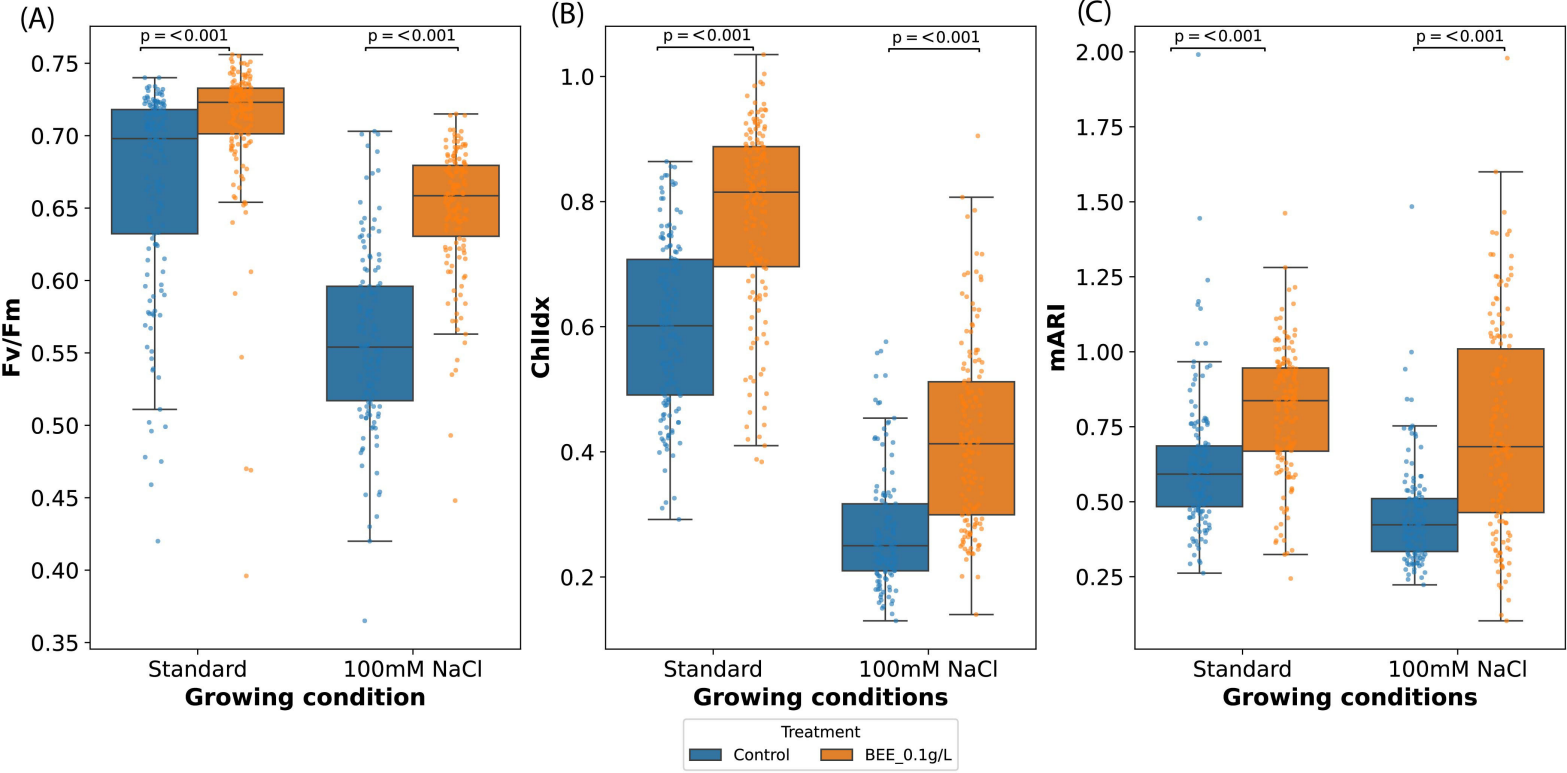
Health parameters of *Arabidopsis* treated with BEE under standard and 100 mM NaCl growth conditions. Shown are the effects on chlorophyll fluorescence **(A)**, chlorophyll index **(B)**, and modified anthocyanin reflective index **(C)**. Data represent the average of 3 biological replicates and 50 technical replicates per bar (150 seedlings in total, 50 seedlings per replicate). Statistical significance (n = 150, *p* < 0.05) between control and BEE treatment in respective groups (growth condition) was performed using two-way ANOVA followed by Tukey’s HSD test.

The effect of BEE on *Arabidopsis* shoot health was evaluated by measuring chlorophyll fluorescence (Fv/Fm), chlorophyll index (ChlIdx), and anthocyanin accumulation (mARI). BEE-treated plants showed a slightly higher Fv/Fm ratio than control plants (0.71 and 0.67, respectively). Under mild salinity stress, BEE treatment sustained a higher Fv/Fm ratio compared with the control (0.65 versus 0.56; **Figure 3A**). The chlorophyll index, a quantification of leaf greenness, showed that BEE-treated plants contained a significantly higher chlorophyll content under both standard and salt-stress conditions (**Figure 3B**). Similarly, anthocyanin levels were significantly higher in BEE-treated plants. (**Figure 3C**).

### 3.2 Effect of BEE on vegetative growth

#### 3.2.1 *Arabidopsis* grown on substrate

**Figure 4:**
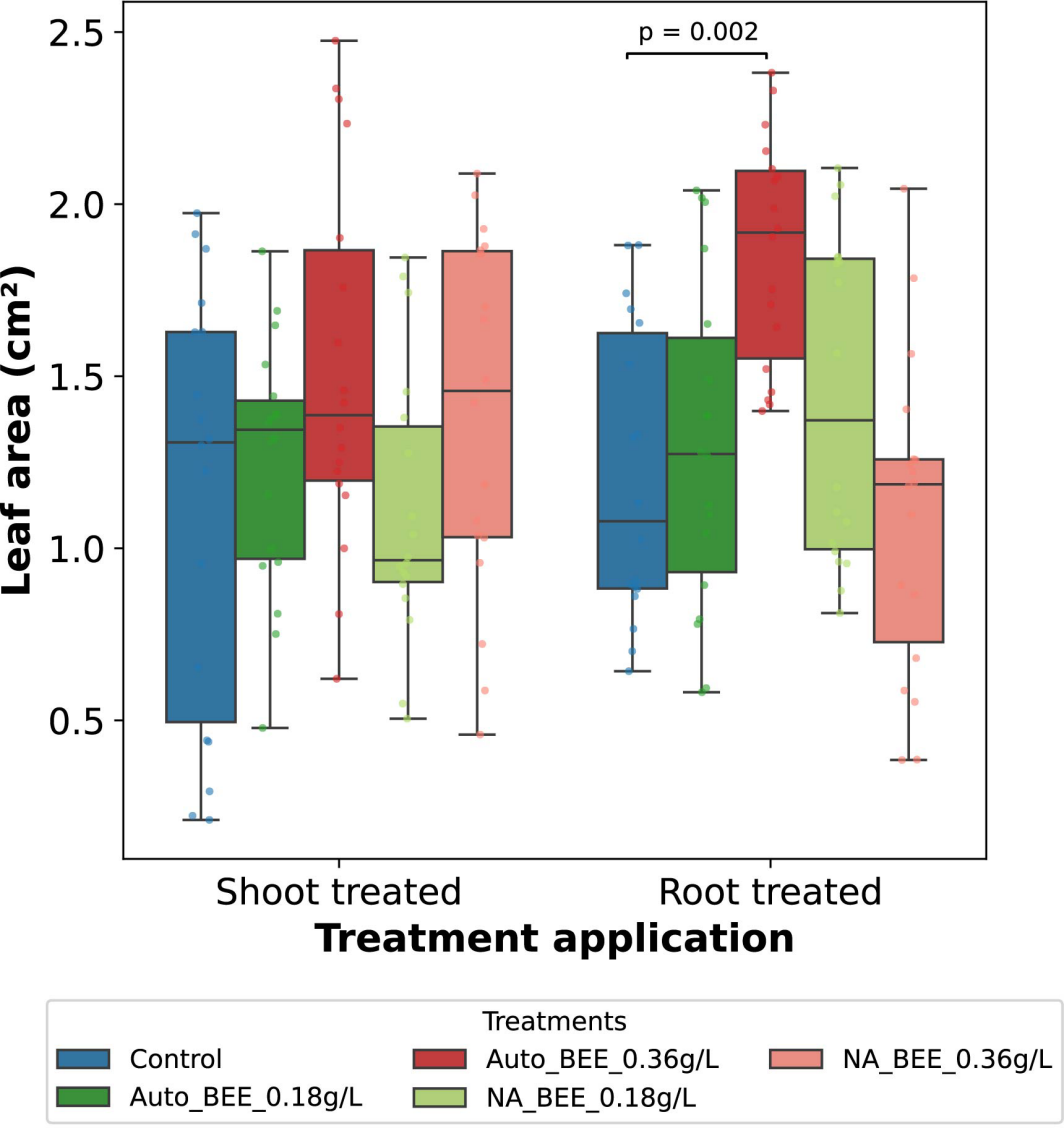
Effect of different forms of BEE and different application methods on *Arabidopsis* leaf area. The data represent 18 technical replicates per bar. Data are presented in a boxplot with all data points and bars indicating the minimum and maximum values. Statistical significance (n = 18, *p* < 0.05) between control and treatment BEE in respective groups (application) are shown and determined using two-way ANOVA followed by Tukey’s HSD test. Auto_BEE, autoclaved Belgian endive extract; NA_BEE, non-autoclaved Belgian endive extract.

In a subsequent series of experiments, we evaluated whether the growth stimulation effect of BEE observed in *in vitro-*cultured *Arabidopsis* could be reproduced in soil conditions. *Arabidopsis* seedlings at 14DAS were treated with 0.18 g/L or 0.36 g/L BEE either by drenching (root treatment) or spraying (shoot treatment). To allow comparison with the *in vitro* test conditions, autoclaving of BEE (Auto_BEE) was required, and untreated (non-autoclaved; NA_BEE) BEE was included as a reference. At 0.18 g/L, neither BEE solution altered the leaf area, suggesting that the shoot growth stimulation observed in the *in vitro* experiments was not reproduced (**Figure 4**). In contrast, the 0.36 g/L autoclaved BEE solution applied by root drenching significantly increased the leaf area, whereas spraying the same solution did not exert growth stimulation (**Figure 4**). These observations support the biostimulant effect of BEE and suggest that its activity increases by autoclaving. Furthermore, it suggests that it is active through the root system.

#### 3.2.2 Lettuce cultivation with BEE

**Figure 5:**
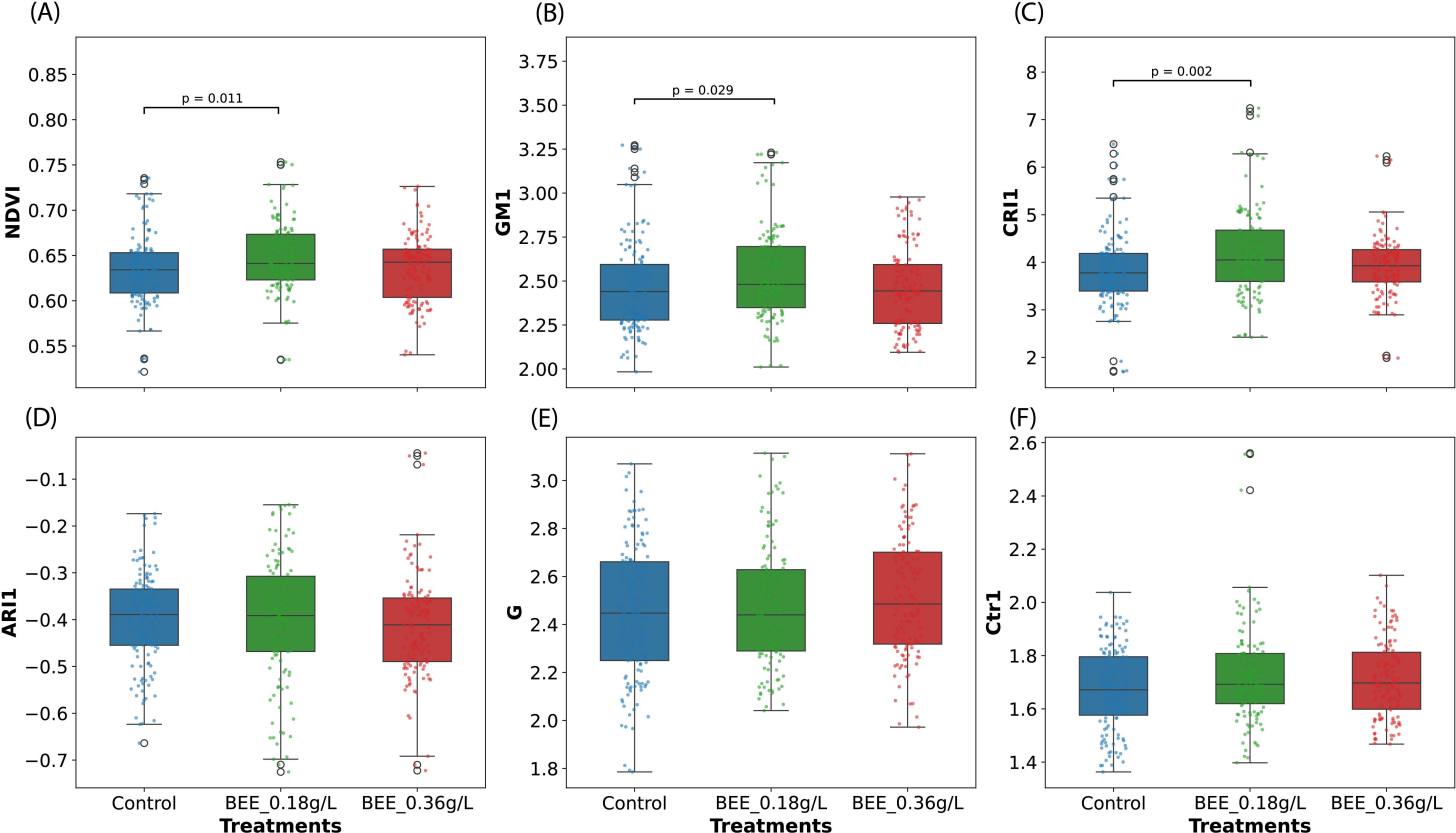
Effect of BEE treatment or control treatment on lettuce leaf optical indices. Shown are **(A)** NDVI, Normalized Difference Vegetation Index; **(B)** GM1, Gitelson and Merzlyak Index 1; **(C)** CRI1, Carotenoid Reflectance Index 1; **(D)** ARI1, Anthocyanin Reflectance Index 1; **(E)** G, Greenness Index; and **(F)** Ctr1, Carter Index 1. *p*-values < 0.05 according to one-way ANOVA followed by Dunnett’s multiple comparison test are displayed on the graphs.

Lettuce seedlings treated with 0.18 g/L or 0.36 g/L BEE were evaluated 31 days after treatment (DAT). The mean shoot weight was compared with that of untreated (control) plants. The BEE-treated plants did not show a significant difference in leaf fresh weight (**Supplementary Figure 4A**) or dry weight (**Supplementary Figure 4B**) relative to the untreated control. The NDVI optical properties of lettuce leaf samples were higher in plants treated with 0.18 g/L BEE (*p* = 0.021) compared with the control, whereas the higher BEE dose (0.36 g/L) did not show a significant effect (**Figure 5A**). The chlorophyll content (GM1) was significantly higher (*p* = 0.029, **Figure 5B**) with 0.18 g/L BEE treatment compared with the control, but not with the 0.36 g/L BEE treatment. The carotenoid content (CRI1 index) was also significantly higher after 0.18 g/L BEE treatment (*p* = 0.002, **Figure 5C**) but not with the 0.36 g/L BEE treatment. No significant differences were observed in anthocyanin content (ARI1), greenness index (G), and the Carter index (Ctr1; **Figures 5D–F**).

### 3.3 Effect of BEE on sweet pepper growth and fruit development

#### 3.3.1 Impact of BEE on sweet pepper grown in a greenhouse

**Figure 6:**
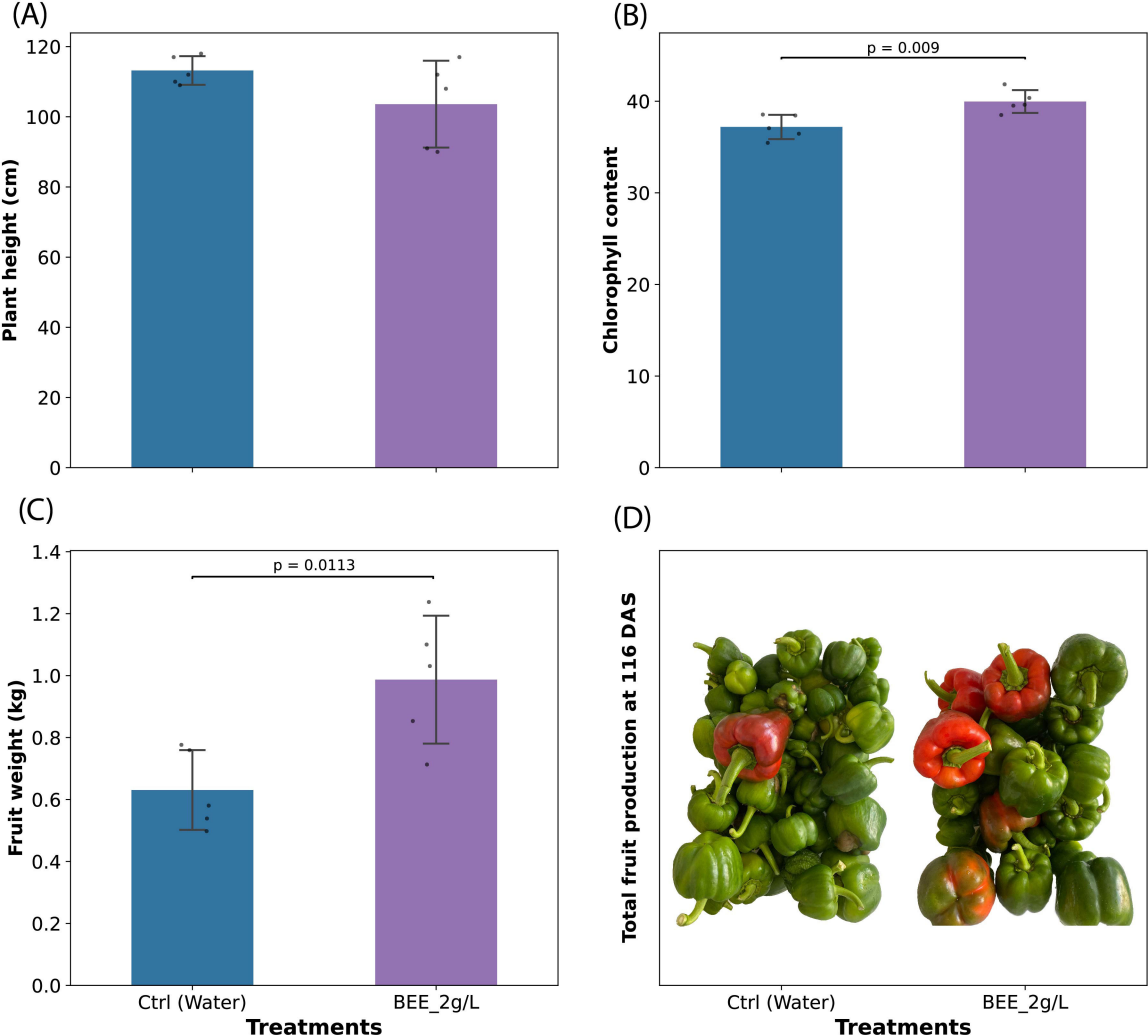
Effect of BEE treatment or control treatment on growth parameters and yield of sweet pepper plants under greenhouse conditions. **(A)** The average plant height. **(B)** The chlorophyll content from DUALEX readings. **(C)** The total fresh weight of all fruits per treatment. **(D)** Pictures comparing the mature fruits from the treated and untreated groups. All graphs are presented with error bars indicating standard deviation. Statistical significance (n = 5, *p* = 0.05) was determined using a t-test. BEE, Belgian endive extract. Ctrl: control treated with water. DAS: days after sowing.

Sweet pepper experiments were conducted in a greenhouse over the course of three consecutive years. In the first round of tests (year 2022; Experiment 5.1), the sweet pepper plants treated with BEE were similar in size at the end of the experiment compared with the control group, reaching an average height of 113.2 ± 4.1 cm and 103.6 ± 12.4 cm, respectively (**Figure 6A**). DUALEX measurements indicated that the chlorophyll content in BEE-treated plants was significantly higher (40.0 µg/cm^2^, *p* = 0.009) compared with the control plants (37.2 µg/cm^2^; **Figure 6B**). This result suggests that BEE-treated plants possess higher photosynthetic activity, which may lead to higher capacity of carbon dioxide fixation.

Pepper plants treated with BEE showed a 56% increase in yield (0.99 ± 0.2 kg of fruits per plant) compared with the control (0.63 ± 0.1 kg of fruits per plant) at 116 DAS (**Figure 6C**). In addition, BEE-treated plants produced fruit that matured earlier than that of the untreated control (**Figure 6D**).

#### 3.3.2 Impact of BEE on sweet pepper grown indoors under artificial light

**Figure 7:**
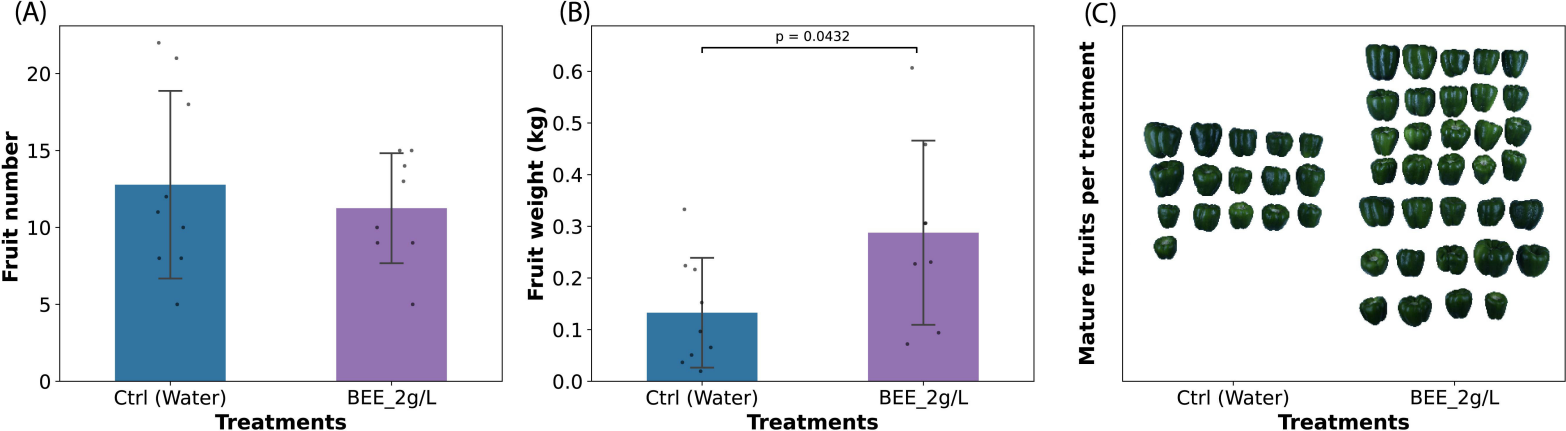
Effect of BEE treatment or control treatment on the yield of sweet pepper plants grown under growth room conditions. **(A)** The average fruit number (Ctrl n = 8; BEE n = 9). **(B)** The total fresh weight of all fruits per treatment. **(C)** Pictures comparing the mature fruits from the treated and untreated groups. All graphs are presented with error bars indicating standard deviation. Statistical significance (n = 8–9, *p* = 0.05) was determined using a t-test. BEE, Belgian endive extract; Ctrl, control treated with water.

To test whether the effect of BEE on sweet pepper fruit development and maturation could be replicated under artificial light conditions, plants were cultured in a controlled environment, a plant growth room (year 2023; Experiment 6). In this trial, young sweet pepper plants were transplanted onto rockwool slabs, and fruit production was evaluated at 97 DAS by counting the number of fruits per plant from treated and non-treated plants and comparing their weights. The average number of fruits per plant, including both young and mature fruits, was 11.3 (± 3.6) per plant in the BEE-treated group, whereas the control group had 12.8 (± 6.1) fruits per plant (**Figure 7A**). This corresponded to a 13.3% higher fruit number in the control group compared to the BEE-treated plants, although the difference was not statistically significant. A significant effect was observed for fruit yield. BEE-treated plants produced an average of 0.29 (± 0.2) kg of fruits per plant, compared with 0.13 (± 0.1) kg of fruits per plant in the control group (*p* = 0.043), corresponding to a 123% increase. Although the average number of fruits per plant in the control group was 13.3% more than in the BEE-treated group, the number of mature fruits in the BEE-treated group (34 fruits) was twice that of the mature fruits in the control group (16 fruits; **Figure 7C**). These results further support the growth-promoting effect of BEE on sweet pepper fruit yield and maturity.

#### 3.3.3 The fruit development effect of BEE

**Figure 8:**
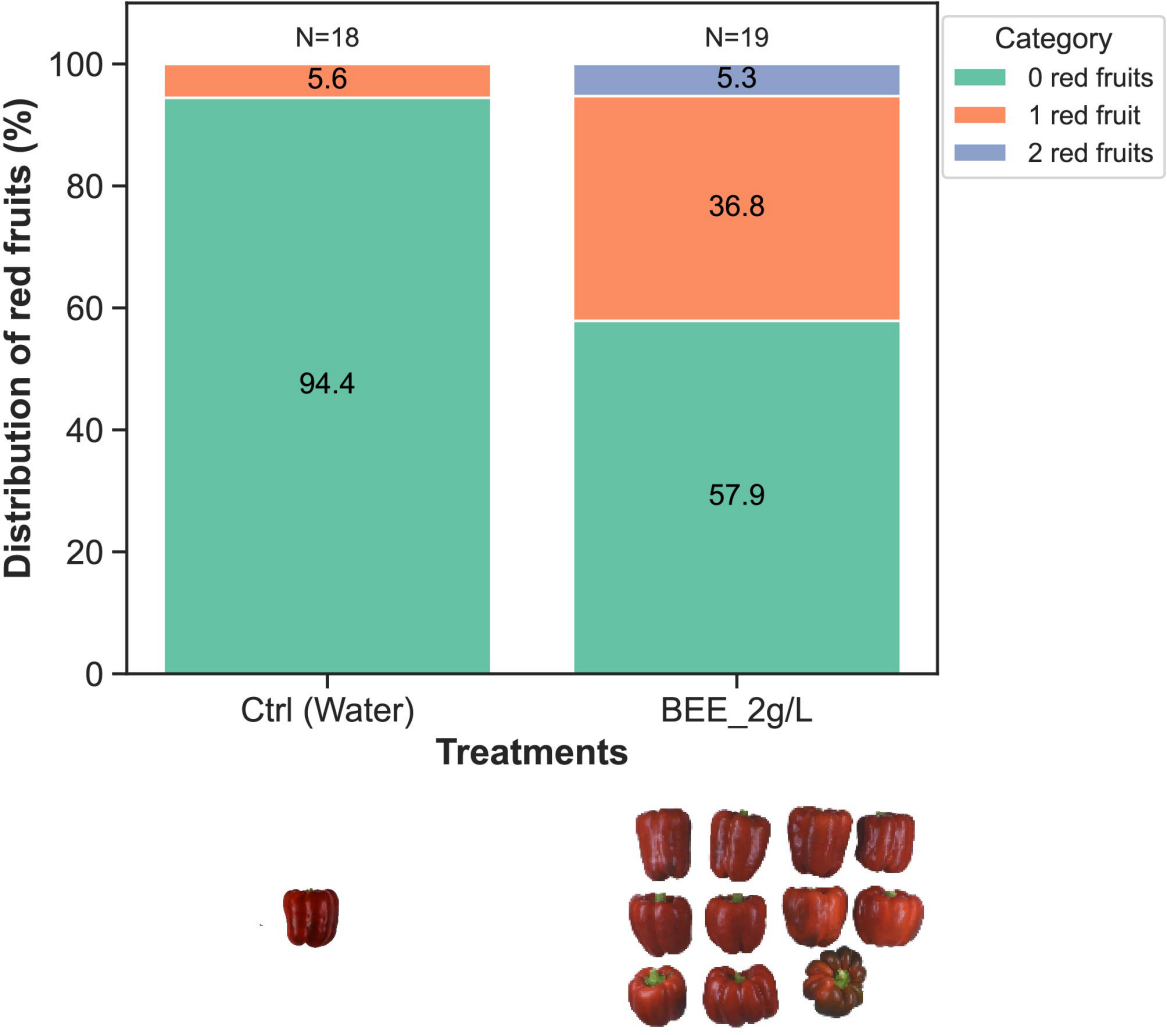
Effect of BEE treatment or control treatment on the maturity and ripeness of sweet pepper fruits under greenhouse conditions. Stacked graph of the percentage distribution of ripe (red) fruits per plant per treatment (top). Pictures comparing the ripe (red) fruits from the treated and untreated groups (bottom). BEE, Belgian endive extract; Ctrl, control treated with water.

The third pepper experiment (year 2024; Experiment 7.1) included 30 plants that were grown in a greenhouse, and fruits were analyzed at 116 DAS. The lower branches of the plants were pruned before flowering to promote fruit quality. As in the previous experiments, BEE treatment consistently enhanced fruit development, producing larger fruit that ripened earlier. The number of red (ripe) fruits and the distribution of mature fruits per plant were determined (**Figure 8**). The BEE-treated plants produced more red fruits (0.47 ± 0.6) than the control plants (0.06 ± 0.2), consistent with the effects of BEE on sweet pepper fruit ripeness and yield observed in previous experiments (Sections 3.2.1 and 3.2.2). A chi-square test showed a significant difference in the distribution of red fruits between groups (χ² = 6.76, df = 2, *p* = 0.034). This difference was mainly driven by a higher proportion of plants with one or more red fruits in the treatment group and a higher proportion of plants without red fruits in the control group. However, the presumed earlier maturation induced by BEE was not reflected in significant differences in plant height, chlorophyll content, fruit number, or fruit weight (**Supplementary Figures 6A–D**).

As fruit ripening is associated with ethylene signaling (Huang et al., 2024), ethylene levels were analyzed in young leaves of 54-day-old plants. BEE-treated plants released a similar level of ethylene as non-treated plants 48 h after treatment (**Supplementary Figure 7**).

### 3.4 Effect of BEE treatment on sweet pepper under drought stress

**Figure 9:**
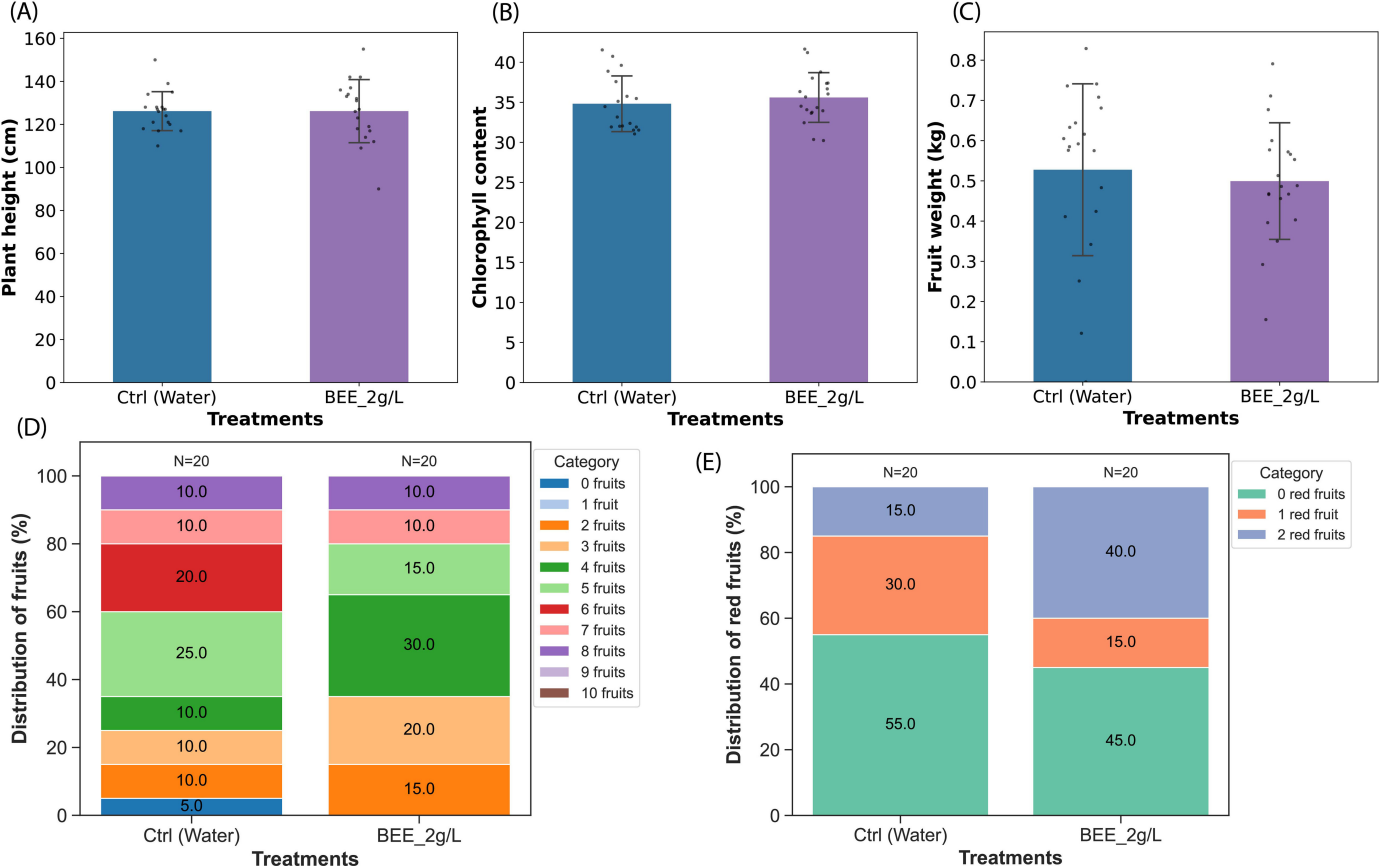
Effect of BEE treatment or control treatment on growth parameters and yield of drought-stressed sweet pepper plants under greenhouse conditions in 2024 (Experiment 7.2). **(A)** The average plant height. **(B)** The chlorophyll content from DUALEX readings. **(C)** The total fresh weight of all fruits per treatment. **(D)** Percentage distribution of fruit number per treatment. **(E)** Percentage distribution of the number of ripe (red) fruits per plant per treatment. Graphs are presented with error bars indicating standard deviation (n = 18–19). Statistical significance (*p* = 0.05) was determined using a t-test.

Greenhouse-grown sweet pepper cultures (year 2024; Experiment 7.2) were subjected to drought stress by interrupting irrigation from Day 58 to Day 62, when the soil moisture reached <30%. BEE was applied on Day 63. Plant height, chlorophyll content, fruit weight, and fruit number were not affected by BEE treatment (**Figures 9A–D**). However, the number of ripe (fully red colored) fruits was much higher in the treated plants, resulting in a higher mean percentage of ripe fruits in the BEE-treated group (30.85%) than in the untreated control group (16.95%), although this difference was not statistically significant. The chi-square test did not reveal a statistically significant difference in the distribution of red fruits between groups (χ² = 3.47, df = 2, *p* = 0.176). However, the observed distributions suggested a trend toward higher frequencies of plants bearing two red fruits in the treatment group (**Figure 9E**). A similar trend was observed in another experiment (year 2022; Experiment 6.2), in which BEE treatment was applied before the drought induction and increased only fruit maturity (**Supplementary Figure 8**). These findings suggest that, although BEE does not appear to directly enhance vegetative growth under drought stress, it tends to promote fruit maturity and ripeness.

## 4 Discussion

In this study, we conducted a series of experiments to investigate the potential beneficial activity of BEE under stress conditions and to assess its applicability in crop production, specifically in lettuce and sweet pepper.

### 4.1 Effect of BEE on abiotic stress

Salinity stress negatively impacts plant growth by inhibiting seed germination, stunting root and shoot development, and ultimately reducing yield. Biostimulants can mitigate these effects by promoting root growth and biomass accumulation (Alzate Zuluaga et al., 2022; Zuzunaga-Rosas et al., 2023; Ntanasi et al., 2024). Plant extract-derived biostimulants, such as protein hydrolysates, have been shown to improve biomass production under salinity stress in crops such as maize (Ertani et al., 2013; Colla et al., 2014). In this study, the biostimulant BEE had no effect on germination under salt stress but mitigated stress-related effects, as evidenced by early true leaf emergence, larger leaf area, and improved plant health compared with untreated plants. Two explanations are possible: (1) BEE actively enhances the plant’s stress tolerance, or (2) BEE promotes early plant vigor, resulting in more developed plants that better tolerate salt stress. As BEE was applied prior to germination and did not affect the germination rate, it appears to directly protect against salt stress rather than through an early growth advantage. BEE enhanced photosynthetic efficiency, thereby contributing to increased biomass and leaf area. Since leaves serve as the primary sites of photosynthesis (Lv et al., 2023), the enhanced effect of BEE on early true leaf emergence under stress may confer an advantage to the photosynthetic performance of the stressed plants, giving them an edge over untreated controls. This improvement may, in turn, promote larger leaf area, creating a positive feedback cycle that further enhances growth under stress conditions.

Salinity stress affects photosynthesis by accelerating chlorophyll degradation and inducing stomatal closure, thereby limiting CO_2_ intake. Biostimulant application has been reported to reduce chlorophyll degradation while enhancing nutrient uptake and maintaining plant productivity (Zuzunaga-Rosas et al., 2022; Ikuyinminu et al., 2023). BEE treatment improved chlorophyll fluorescence and chlorophyll index, contributing to overall plant health under salt stress. Anthocyanins are photo-protectants that can enhance chlorophyll fluorescence, chlorophyll index, and leaf expansion (Steyn et al., 2002; Gould, 2004). Under standard conditions, the increased anthocyanin accumulation in BEE-treated plants is unlikely to indicate oxidative stress, as overall plant health and growth parameters remained unaffected. However, mARI may still function as a photo-protectant, particularly in young plants, as Fv/Fm values were only slightly above the 0.7 threshold, suggesting that the spectral measurements might have detected a mild stress response in developing seedlings. Taken together, these findings suggest that BEE treatment enhances *Arabidopsis* health by improving photosynthetic efficiency, chlorophyll content, and anthocyanin accumulation, particularly under mild salt stress. The higher Fv/Fm values, chlorophyll index, and mARI suggest BEE mitigates stress effects while potentially acting as a photo-protectant in young seedlings. These findings highlight BEE’s potential in enhancing plant resilience and tolerance to salinity stress. Additional benefits of biostimulants in mitigating salinity stress include enhanced antioxidant activity (Li et al., 2022a; Naboulsi et al., 2022), improved ion homeostasis, and better osmotic regulation (D’amato and Del Buono, 2021). The results from this study align with the established role of biostimulants in alleviating salt stress.

In addition to salt toxicity, we analyzed osmotic (drought) stress by transferring seedlings to media containing 100 mM or 150 mM sorbitol. There was no consistent significant alleviation of osmotic stress under *in vitro* conditions, nor did we observe an advantage of BEE treatment in plants subjected to drought stress. Similarly, *ex vitro* experiments confirmed that BEE did not enhance growth parameters in drought-stressed sweet pepper plants compared with controls. These findings contrast with previous studies that reported that biostimulants can mitigate drought stress by improving growth and yield (De Clercq et al., 2023), enhancing photosynthetic efficiency (Soussani et al., 2023), and optimizing nutrient and water-use efficiency (Repke et al., 2022).

Overall, these results suggest that BEE does not mitigate abiotic stress by reducing osmotic pressure. Instead, its beneficial effects appear to be associated with improved photosynthetic efficiency and biomass accumulation. The potential effects of BEE on metabolic processes and stress-related signaling pathways were not addressed in the present study.

### 4.2 Evaluation of BEE bioactivity *ex vitro*

The use of both model plants and economically important crops in biostimulant research is essential for bridging fundamental and applied studies. Model plants, such as *A. thaliana*, provide critical insights into molecular pathways, gene expression, and physiological responses triggered by biostimulants. In contrast, crops and horticultural plants validate these findings under field conditions, ensuring practical applicability for large-scale agriculture (Rouphael and Colla, 2020). This dual approach strengthens the scientific foundation of biostimulant research while promoting sustainable agricultural practices. In the *in vitro* experiments, BEE treatment significantly increased leaf area in *Arabidopsis*, serving as a key indicator of shoot growth. Given this significant growth enhancement, it was necessary to evaluate whether these effects could be replicated under *ex vitro* conditions in both *Arabidopsis* and crop plants. Initial *ex vitro* experiments in *Arabidopsis* tested two forms of BEE, as *in vitro* applications typically involve autoclaving BEE together with the growth medium. When BEE was not autoclaved in an *in vitro* experiment, some treated plants failed to outperform the controls, while others showed contamination (**Supplementary Figure 9**). This observation suggests that heat treatment enhances the bioactivity of BEE, with heat-treated BEE demonstrating the strongest effects on both vegetative and reproductive growth.

Translating *in vitro* findings to *ex vitro* conditions also requires an understanding of how plants absorb treatments. In *in vitro* experiments, BEE is uniformly available to both root and shoot tissues in contact with the medium, whereas under *ex vitro* conditions, uptake depends on the application method. In the present study, clear biostimulant effects were observed after root drenching, while no effect was detected following shoot spraying. Root drenching typically enhances root development and nutrient uptake (Colla et al., 2015; Lucini et al., 2015, 2018), while foliar application targets photosynthesis, metabolism, and stress responses (Lucini et al., 2015; Cristiano et al., 2018; Peroni et al., 2024). Although root drenching was the most effective method for BEE application, a combination of root and foliar application may provide additional benefits, as suggested by the *in vitro* experiments and previous reports (Lucini et al., 2015).

The initial *ex vitro* results revealed insights into the application strategy of BEE in crops. Lettuce was used as a model leafy vegetable to assess the effect of BEE on its growth. In this experiment, heat-treated BEE was applied via root drenching, and no significant differences were observed in shoot fresh and dry weights between the BEE-treated and control plants. Leaf area, which was measured in other experiments, could not be assessed here due to the characteristics of the Expertise variety, hence shoot weight was assessed. The comparable differences in shoot fresh and dry weights indicate that the similarity in fresh weight was not due to differences in water retention, suggesting that BEE did not influence leaf water retention. BEE treatment, however, promoted plant greenness and chlorophyll content, as evidenced by significantly higher leaf indices (NDVI and GM1) associated with photosynthetic activity and general plant health. This interpretation is supported by reflectance studies demonstrating that chlorophyll content is closely linked to plant physiological status and can be assessed non-destructively using spectral indices, including NDVI and Gitelson–Merzlyak-type chlorophyll indices (Gitelson et al., 2003). These observations are consistent with the positive effects of BEE on plant performance and health under salt-stress observed in this study (Section 3.1.2). Moreover, similar biomass-neutral but physiology-positive responses have also been reported in green lettuce cv. Expertize RZ, where a plant-derived biostimulant from borage (*Borago officinalis*, L.) did not affect head weight but improved nitrogen-flavonol and Fv/Fm ratio (Franzoni and Ferrante, 2024). In addition, BEE increased carotenoid content, which is physiologically relevant because carotenoids are essential for photosynthesis, photoprotection, and pigment balance in plants (Sun et al., 2022). Similar increases in carotenoid/photosynthetic pigment pools have been reported following biostimulant treatment in lettuce, including studies using root drenching (Puglisi et al., 2022).

Previous studies have shown that plant-derived biostimulants increased leaf biomass in the short term and enhanced reproductive traits, including fruit number and fruit weight, in *Capsicum* spp. in the long term (Ertani et al., 2014). BEE significantly increased fruit yield (weight) in two consecutive years but not in the third year. While cultivation practices differed across the three pepper experiments, the decline in efficacy of BEE observed in the third year is likely attributable to the long-term storage of BEE and associated slow decline in bioactivity. Variability in performance has been reported for some biostimulants. For instance, Ertani et al. (2015) reported that grape skin extract and alfalfa hydrolysates both increased fruit quality in chili pepper, but differed in effectiveness due to their origin, processing methods, and endogenous hormone content, including indole-3-acetic acid (Ertani et al., 2015). More broadly, different groups of biostimulants, including plant and algae extracts, protein hydrolysates, and humic substances, have been shown to enhance fruit traits and quality such as antioxidant capacity, ascorbic acid, carotenoids, and capsaicinoids across multiple crops (Rodrigues et al., 2020). These studies emphasize that biostimulant responses are product- and cultivar-specific, necessitating targeted optimization strategies.

A key trait of BEE treatment that remained consistent was the acceleration of fruit ripening. Similar observations have recently been reported in pepper treated with *Ascophyllum nodosum* extract, where foliar application at flowering increased first-harvest yield and fruit number. The authors interpreted the reduced second harvest as evidence that a larger portion of the first fruit set had grown and matured faster (Staykov et al., 2025). Although ethylene is a central regulator of many fruit ripening processes, *Capsicum* is generally considered non-climacteric, with limited ethylene production and low 1-aminocyclopropane 1-carboxylate (ACC) and ACC synthase (ACS) activity during ripening (Aizat et al., 2013; Tipu and Sherif, 2024). However, some pepper cultivars exhibit climacteric-like ripening behavior when attached to the plant (Hou et al., 2018). Therefore, the absence of a significant ethylene increase within 48 h of BEE application indicates that BEE does not trigger an immediate climacteric-like ethylene spike. Instead, the observed effects may result from indirect modulation of developmental pathways, potentially through improved fruit set, source–sink metabolism, or priming of metabolic processes that later influence ripening dynamics. This interpretation is supported by studies showing that biostimulants can increase fruit set, fruit number, yield, sugar accumulation, and metabolic or transcriptomic changes in pepper and sweet pepper (Zamljen et al., 2024; Halshoy and Sadik, 2025; Staykov et al., 2025). It is also consistent with recent evidence indicating that pepper ripening is governed by interacting hormonal, transcriptional, epigenetic, carotenoid, and ABA-related pathways rather than by ethylene alone (Song et al., 2025). More generally, plant biostimulants are increasingly understood to act through complex regulatory networks involving metabolism, enzyme and protein regulation, hormone-like activity, gene expression, and transcriptional pathways rather than through single hormonal pathways (Baltazar et al., 2021; Omoarelojie et al., 2021).

A notable consideration when interpreting biostimulant effects is the time delay between application and phenotypic response. In many cases, this lag phase reflects indirect mechanisms, such as modulation of soil–plant–microbe interactions and gradual shifts in nutrient availability and signaling pathways (Hellequin et al., 2020). Similarly, complex biomolecules such as polysaccharides, e.g., present in seaweed extracts, require (microbial) degradation before becoming active (Hellequin et al., 2020; Andreotti et al., 2022). Additionally, when applied during early developmental stages, biostimulants often act as priming agents that influence downstream processes such as flowering, fruit set, and ripening (Kazakov et al., 2024; Staykov et al., 2025). However, in this study, this delay may have been minimized due to the activation of BEE done by heat treatment before application, which may have increased the immediate availability of active compounds. Furthermore, although studies report cumulative benefits following repeated applications (Staykov et al., 2025), the single application and one-time harvest design used in this study suggest that the observed responses reflect short-term effects, although cumulative impacts cannot be excluded under repeated application scenarios.

Overall, these findings reinforce the importance of considering product stability, biochemical composition, and application timing when evaluating biostimulant performance. Future work should focus on elucidating the mechanisms underlying BEE activity, particularly its interaction with hormonal signaling and developmental processes, along with its stability during storage. Such insights will be essential for optimizing its use and ensuring consistent agronomic outcomes.

## 5 Conclusion

In conclusion, the application of BEE demonstrates significant potential in enhancing various aspects of plant development, including vegetative growth and fruit quality. Furthermore, the results corroborate that the growth-promoting effects of BEE observed *in vitro* can be translated to *ex vitro* cultivation systems. Notably, root application of heat-treated BEE was the most effective strategy, underscoring the importance of both application method and treatment form in maximizing biostimulant efficacy. This study contributes to the growing body of evidence supporting the role of biostimulants in promoting plant vigor, improving nutrient uptake, and increasing tolerance to abiotic stress. It particularly provides a strategy for streamlining the identification of bioactivity in newly discovered biostimulants while highlighting the importance of translating *in vitro* findings to *ex vitro* conditions.

## Data Availability

The raw data supporting the conclusions of this article will be made available by the authors, without undue reservation.
