# Supplementary material for: Belgian endive-derived biostimulant activity in Arabidopsis, lettuce, and sweet pepper at different developmental stages, environmental conditions, and application methods
Source: Front Plant Sci. 2026 Jul 1;17:1852440. doi: 10.3389/fpls.2026.1852440 (PMC13368736; doi:10.3389/fpls.2026.1852440)
Supplement: Supplementary file 1 [file SupplementaryFile1.pdf]

Supplementary file

Table S1. Overview of the spectral parameters and vegetative indices used in this study for the estimation of Arabidopsis health status and lettuce leaf reflectance respectively.

Parameter	Formula	Physiological importance	Source
F_0		Minimal level of fluorescence measured at 730nm* after exposure to a weak measuring beam.	(Baker, 2008; Murchie and Lawson, 2013)
F_m		Maximum level of fluorescence measured at 730nm* after exposure to a brief saturating pulse.	(Baker, 2008; Murchie and Lawson, 2013)
$\frac{F_v}{F_m}$	$\frac{F_m - F_0}{F_m}$	Chlorophyll fluorescence; Efficiency of photosystem II in a dark-adapted state (PSII).	(Baker, 2008; Murchie and Lawson, 2013)
ChlIdx	$\frac{\rho_{770}}{\rho_{710}} - 1$	Chlorophyll index; Vegetation index for the estimation of the chlorophyll content in leaves.	(Gitelson et al., 2003)
mARI	$\left(\frac{1}{\rho_{550nm}} - \frac{1}{\rho_{710nm}}\right) \rho_{770nm}$	Modified Anthocyanin Reflectance Index; Gives an estimation of the anthocyanin-content in leaves.	(Gitelson et al., 2009)
NDVI	$\frac{R_{800} - R_{670}}{R_{800} + R_{670}}$	Normalized Difference Vegetation Index; reflects plant greenness and vigour.	(Rouse et al., 1974)
GM1	$\frac{R_{750}}{R_{550}}$	Gitelson and Merzlyak Index 1; estimates chlorophyll content.	(Gitelson and Merzlyak, 1997)
CRI1	$\frac{1}{R_{510}} - \frac{1}{R_{550}}$	Carotenoid Reflectance Index 1; measures carotenoid/chlorophyll ratio.	(Gitelson et al., 2007b)

ARI1	$\frac{1}{R_{550}} - \frac{1}{R_{700}}$	Anthocyanin Reflectance Indices 1; estimates anthocyanin content.	(Gitelson et al., 2007a)
G	$\frac{R_{554}}{R_{677}}$	Greenness Index; estimates colour.	(Zarco-Tejada et al., 2001)
Ctr1	$\frac{R_{695}}{R_{420}}$	Carter Index 1; estimating plant stress.	(Carter, 1994)

*Spectral width of 40 nm at full width half maximum.

Table 2.2. Experimental setup of sweet pepper experiment, detailing the experimental year, BEE treatment, planting season and substrate.

**Experiment year / Condition**	**Experiment no from Table 2.1**	**Year prepared***	**No of plants per treatment**	**Treatment conc.**	**Planting period / Substrate**
2022 / Greenhouse	5.1, 5.2	2022	10	2g/L	April – July / Potting soil
2023 / Growth room	6	2022	9	2g/L	June – Aug / Rockwool
2024 / Greenhouse	7.1, 7.2	2022	30	2g/L	April – July / Potting soil

*BEE was prepared by autoclaving.

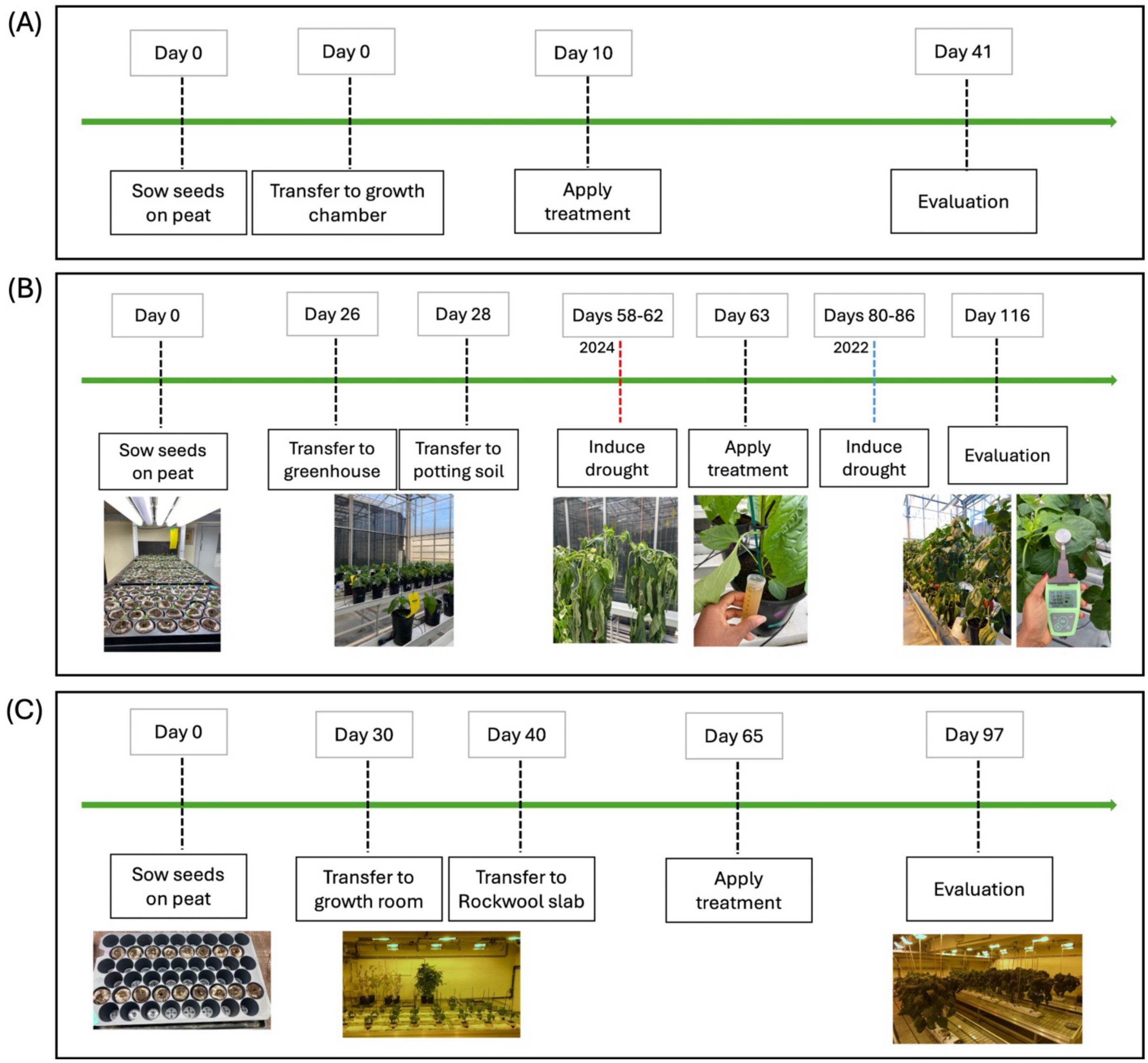

Figure S1. Cultivation scheme of crop experiments from sowing to evaluation. (a) Growth cycle of lettuce grown in a growth chamber. (b) Scheme of greenhouse cultivation of sweet pepper including when drought was induced in 2024 (red dashed line; experiment 7.2) and in 2022 (blue dashed line; experiment 5.2). (c) Growth cycle of sweet pepper grown in the growth room.

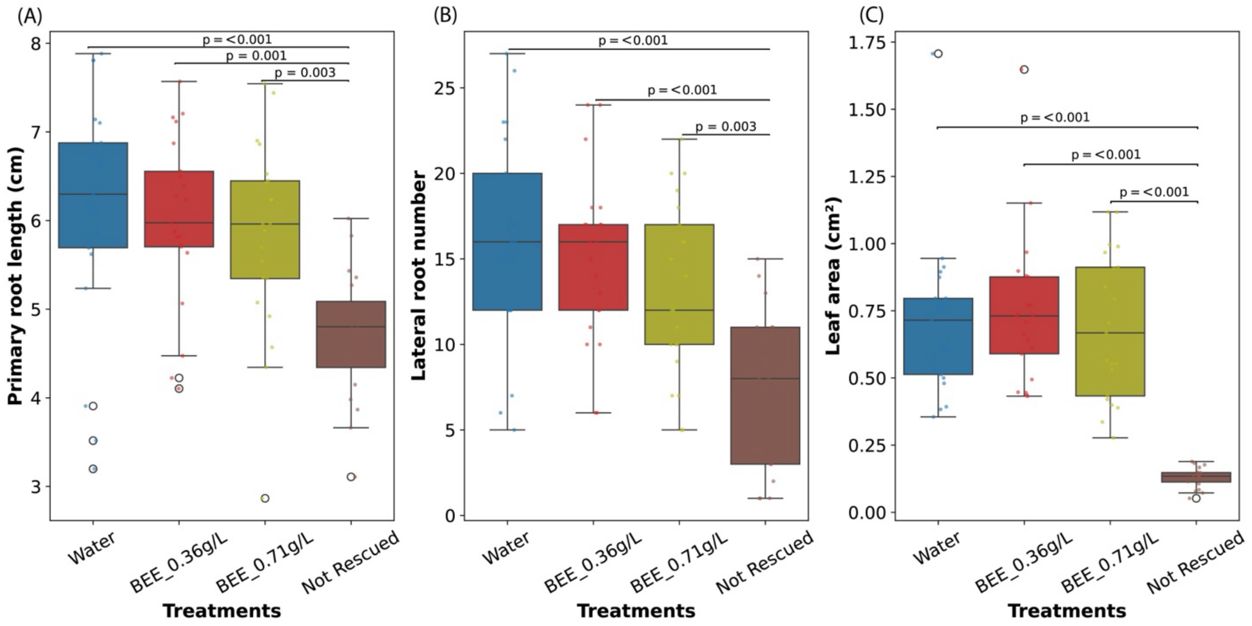

Figure S2. Ability of BEE to rescue osmotic stressed plants. (a) shows the primary root length of plants rescued from 150mM Sorbitol to either BEE (0.36g/Lor 0.71g/L), water (control), or not rescued. (b) and (c) show the effect of BEE on the lateral root and the rosette area respectively. Data represent the average of three biological and seven technical replicates per bar (21 seedlings in total, 7 per replicate). *p*-values indicate significant differences ($p<0.05$) between non-rescued and rescued plants according to Tukey's multiple comparison test.

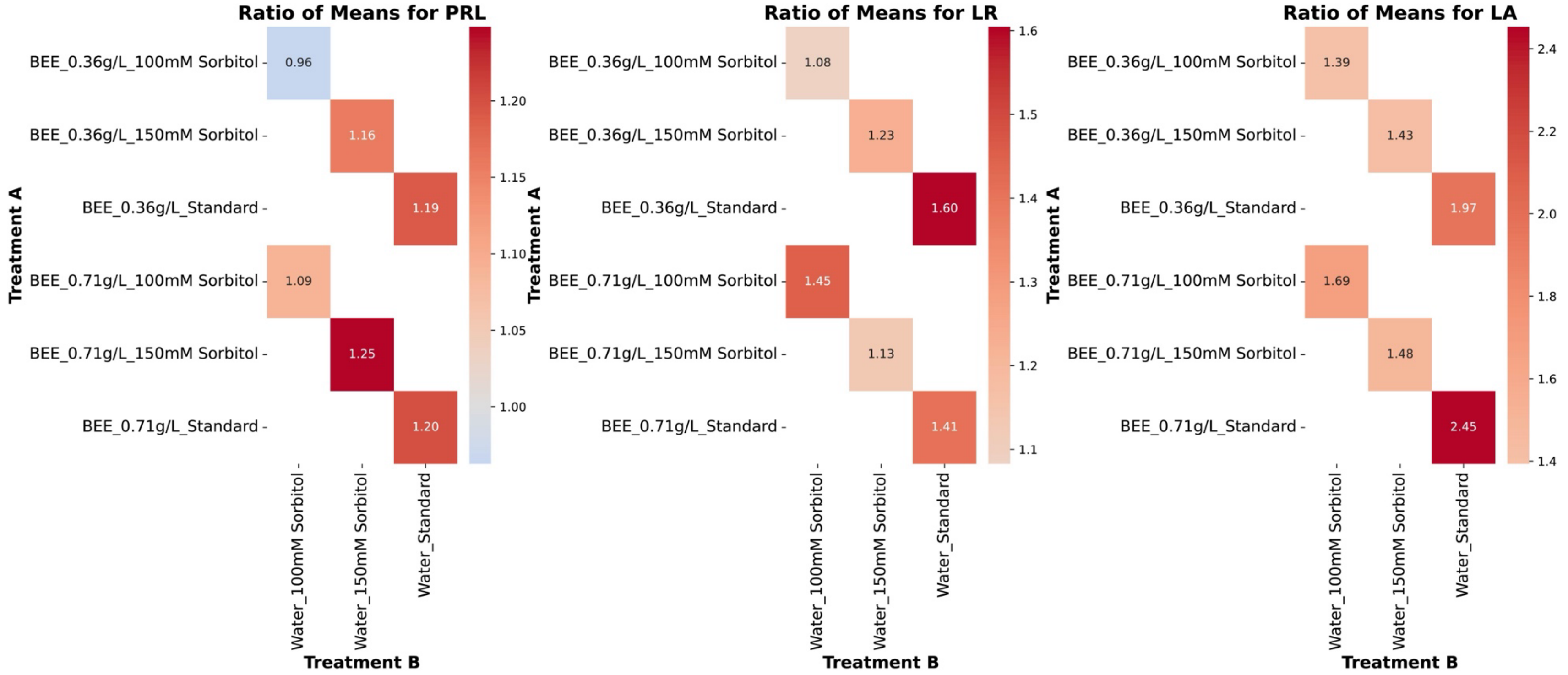

Figure S3. Ratio between BEE application or not in a sorbitol or sorbitol-free medium. Treatment A and B represent BEE treatments and untreated control (water) respectively under standard and osmotic stress conditions. The ratio shows that BEE efficacy diminishes in the root under osmotic stress, except at higher concentration and under mild stress (LR) or high stress (PRL).

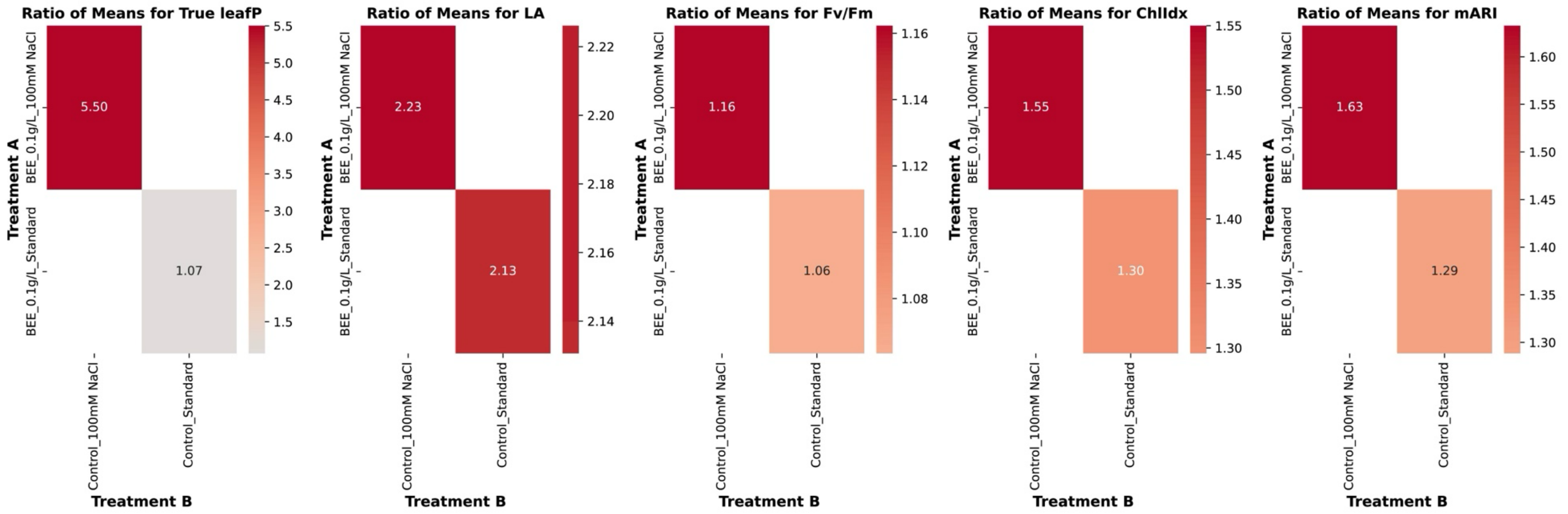

Figure S4. Ratio between BEE application or not in a NaCl or NaCl-free medium. Treatment A and B represent BEE treatments and untreated control (water) respectively under standard and 100 mM NaCl stress. The ratio indicates that BEE is impactful on leaf area under both standard and NaCl conditions and its effect was maximal on the emergence of BEE under NaCl stress.

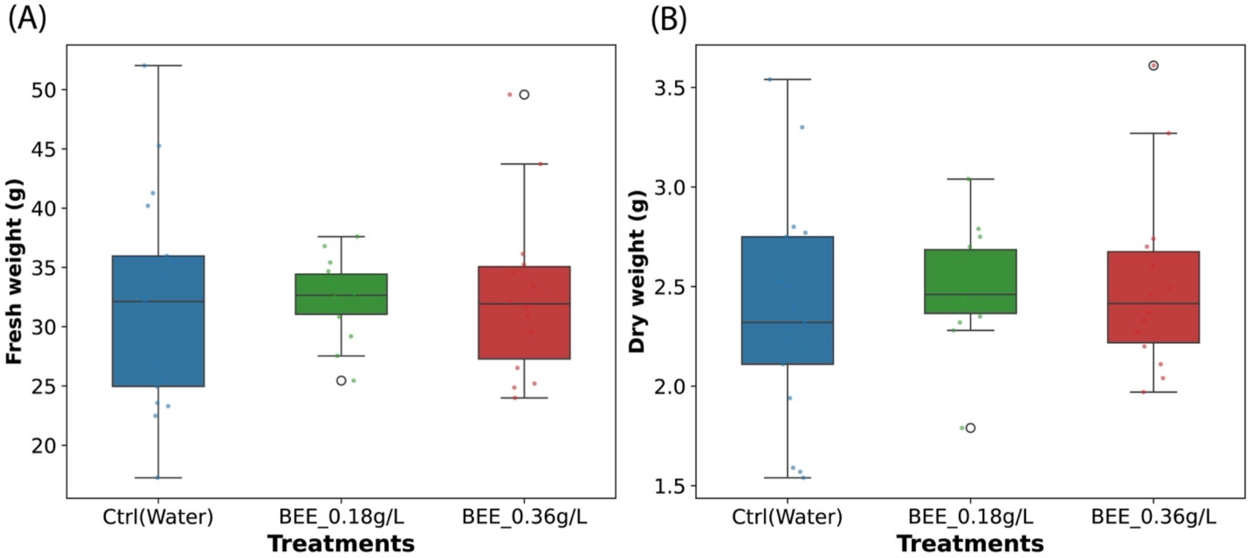

Figure S5. Effect of BEE treatment or not (Ctrl (Water)) on Lettuce fresh (A) and dry (B) weight. Graph is presented in a boxplot with all datapoints overlaid. N= 15-18.

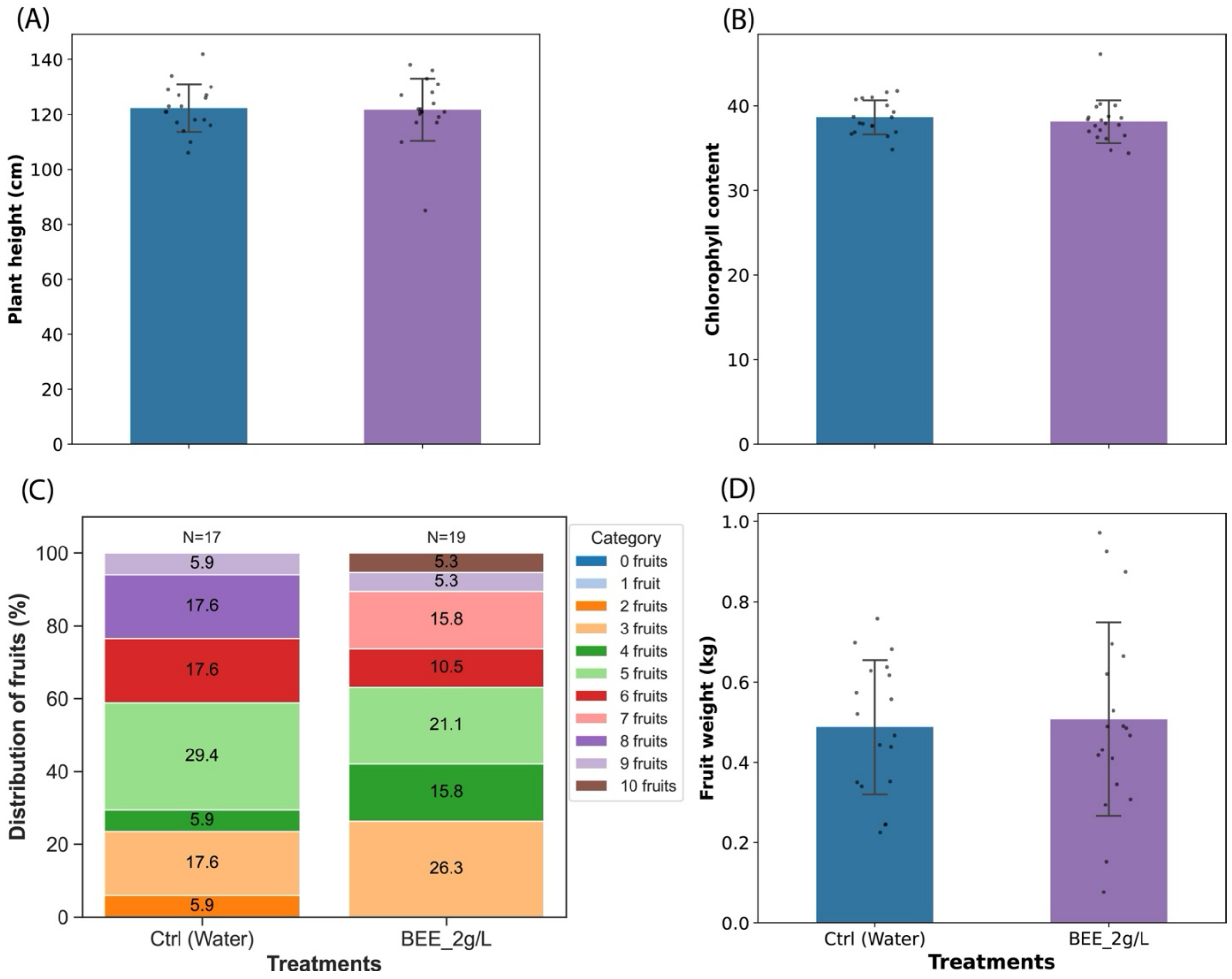

Figure S6. Effect of BEE treatment or not on growth parameters and yield of paprika plants under greenhouse conditions (year 2024; experiment 7.1). (A) The average plant height. (B) The chlorophyll content from DUALEX readings. (C) The percentage distribution of fruits per treatment. (D) The total fresh weight of all fruits per treatment. Graphs are presented with error bars indicating standard deviation (n=17-25). BEE: Belgian endive extract. Ctrl: Control treated with water.

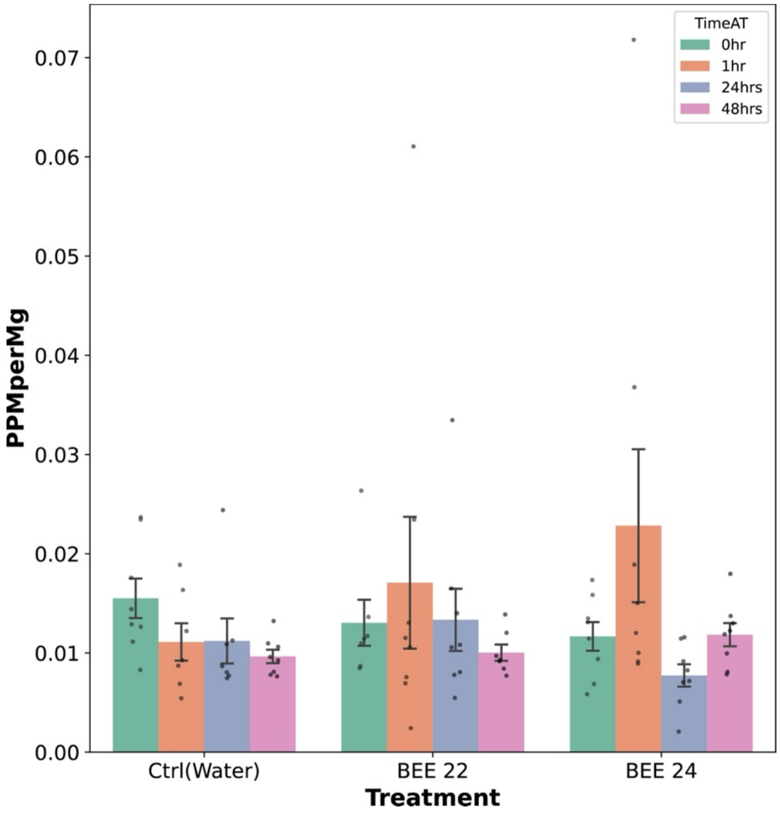

Figure S7. The ethylene quantified from plants treated with BEE or not (Ctrl) for 1 hour, 24 hours, and 48 hours. BEE: Belgian endive extract. Ctrl: Control treated with water. Graph is presented with error bars indicating standard deviation.

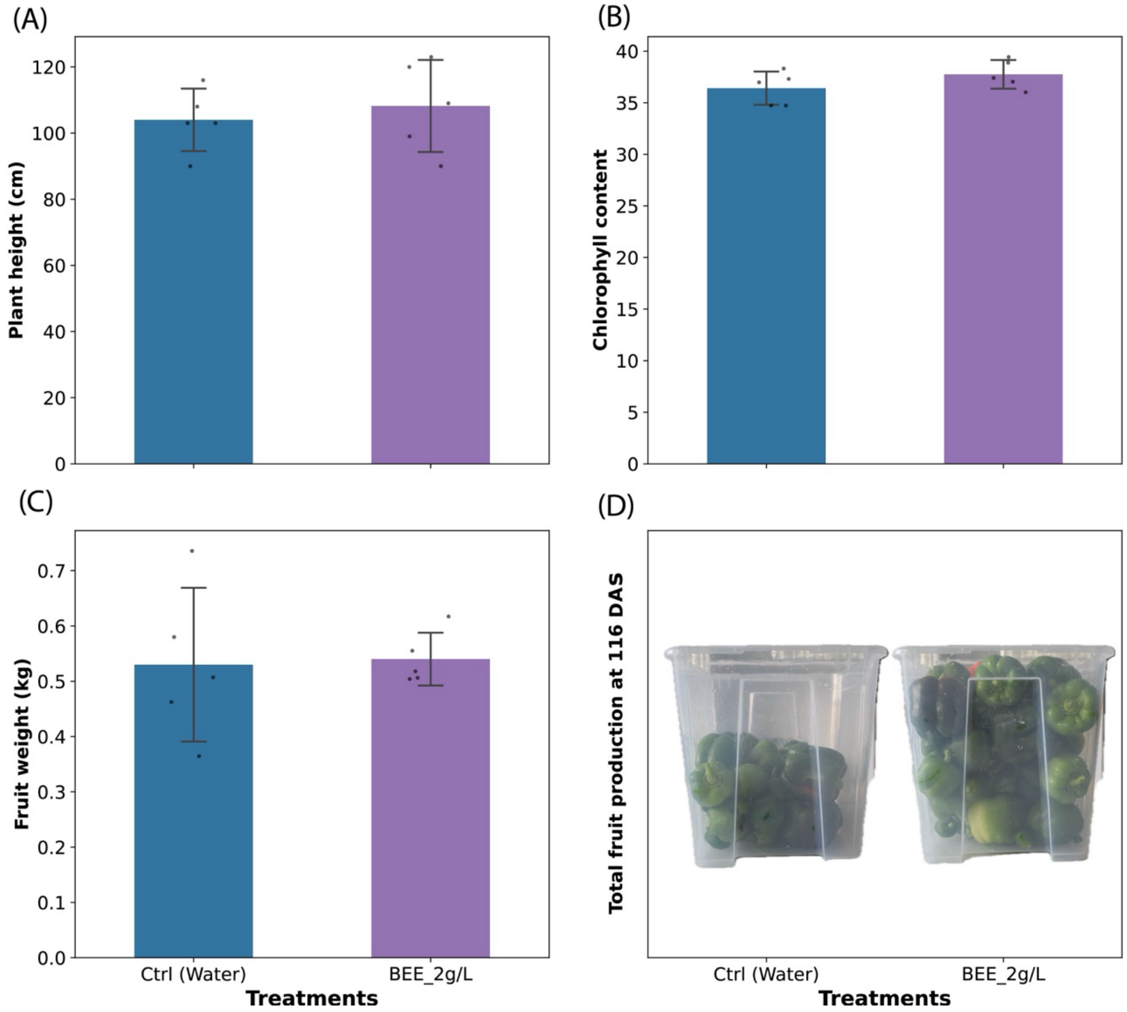

Figure S8. Effect of BEE treatment or not on growth parameters and yield of drought stressed sweet pepper plants under greenhouse conditions in 2022 (experiment 5.2). (A) The average plant height. (n=5-10). (B) The chlorophyll content from DUALEX readings (n=5). (C) The total fresh weight of all fruits per treatment (n=5-10). (D) Pictures comparing the fruit size from treated and untreated groups. All graphs are presented with error bars indicating standard deviation and statistical significance was performed using t-test. BEE: Belgian endive extract. Ctrl: Control treated with water. DAS: Days after sowing.

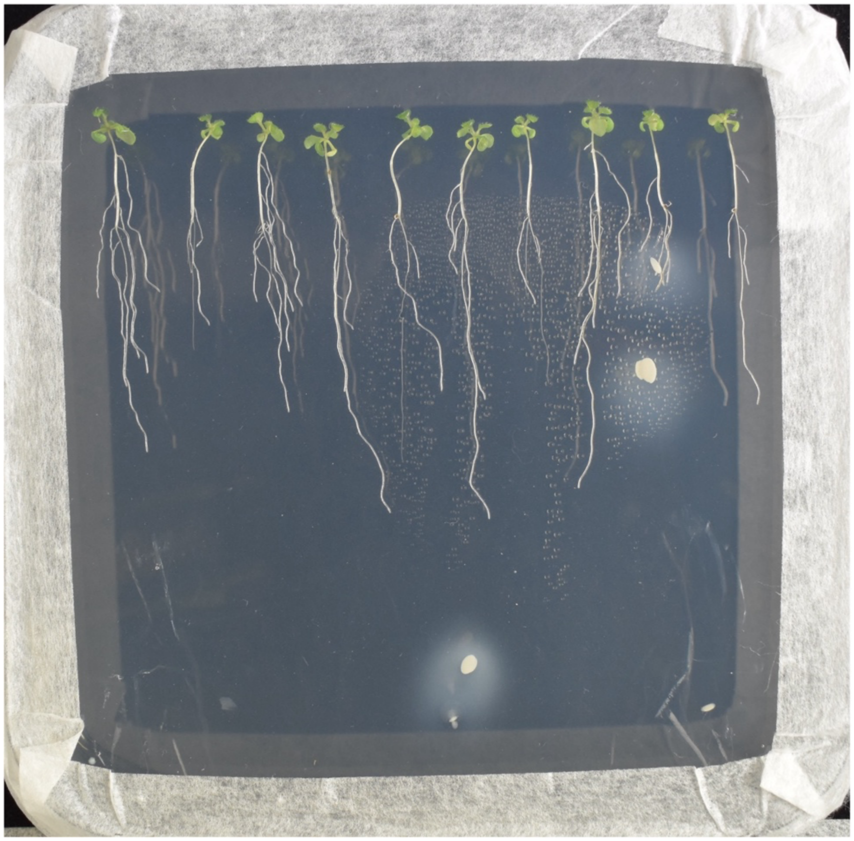

Figure S9. Picture of *Arabidopsis* seedlings treated with non-autoclaved BEE. The treatment caused contamination in the growth medium.

References

Baker, N. R. (2008). Chlorophyll fluorescence: A probe of photosynthesis in vivo. *Annu. Rev. Plant Biol.* 59, 89–113. doi: 10.1146/ANNUREV.ARPLANT.59.032607.092759/CITE/REFWORKS

Carter, G. A. (1994). Ratios of leaf reflectances in narrow wavebands as indicators of plant stress. *Int. J. Remote Sens.* 15, 517–520. doi: 10.1080/01431169408954109

Gitelson, A. A., Chivkunova, O. B., and Merzlyak, M. N. (2009). Nondestructive estimation of anthocyanins and chlorophylls in anthocyanic leaves. *Am. J. Bot.* 96, 1861–1868. doi: 10.3732/ajb.0800395

Gitelson, A. A., Gritz, Y., and Merzlyak, M. N. (2003). Relationships between leaf chlorophyll content and spectral reflectance and algorithms for non-destructive chlorophyll assessment in higher plant leaves. Available at: http://www.urbanfischer.de/journals/jpp

Gitelson, A. A., and Merzlyak, M. N. (1997). Remote estimation of chlorophyll content in higher plant leaves. *Int. J. Remote Sens.* 18, 2691–2697. doi: 10.1080/014311697217558

Gitelson, A. A., Merzlyak, M. N., and Chivkunova, O. B. (2007a). Optical Properties and Nondestructive Estimation of Anthocyanin Content in Plant Leaves. *Photochem. Photobiol.* 74, 38–45. doi: 10.1562/0031-8655(2001)0740038opaneo2.0.co2

Gitelson, A. A., Zur, Y., Chivkunova, O. B., and Merzlyak, M. N. (2007b). Assessing Carotenoid Content in Plant Leaves with Reflectance Spectroscopy. *Photochem. Photobiol.* 75, 272–281. doi: 10.1562/0031-8655(2002)0750272accipl2.0.co2

Murchie, E. H., and Lawson, T. (2013). Chlorophyll fluorescence analysis: A guide to good practice and understanding some new applications. *J. Exp. Bot.* 64, 3983–3998. doi: 10.1093/jxb/ert208

Rouse, J. W., Haas, R. H., Schell, J. A., and Deering, D. W. (1974). Monitoring vegetation systems in the Great Plains with ERTS. Available at: https://ntrs.nasa.gov/citations/19740022592 (Accessed May 15, 2026).

Zarco-Tejada, P. J., Miller, J. R., Noland, T. L., Mohammed, G. H., and Sampson, P. H. (2001). Scaling-Up and Model Inversion Methods with Narrowband Optical Indices for Chlorophyll Content Estimation in Closed Forest Canopies with Hyperspectral Data.
